# Scalable Membrane-Based Lithium-Ion Sieve Sorbent for Selective Lithium Extraction from Brines

**DOI:** 10.1021/acs.langmuir.6c04005

**Published:** 2026-10-04

**Authors:** Ana Ivanković, Adrien Comès, Martin Stockhausen, Justyna Florek, Heinz Ragossnig, Cana Khalouche, Thilo Hofmann, Freddy Kleitz, Christian M. Pichler

**Affiliations:** † 27259 Vienna University of Technology, Institute of Applied Physics, Wiedner Hauptstrasse 8-10, 1040 Vienna, Austria; ‡ 27258 University of Vienna, Department of Functional Materials and Catalysis, Währinger Strasse 42, 1090 Vienna, Austria; § University of Vienna, Vienna Doctoral School in Chemistry (DoSChem), Währinger Strasse 42, 1090 Vienna, Austria; ∥ Amer-Sil S.A., 61 Rue d’Olm, L-8281 Kehlen, Luxembourg; ⊥ University of Vienna, Department of Environmental Geosciences, Josef-Holaubek-Platz (UZA II), 1090 Vienna, Austria; # 344665 Treibacher Industrie AG, Auer-von-Welsbach-Strasse 1, 9330 Althofen, Austria

## Abstract

The escalating global demand for lithium, driven by the rapid growth of production of electric vehicles and portable electronics, requires the development of selective and scalable extraction technologies capable of recovering lithium from complex liquid resources such as geothermal-, oilfield-, or low-grade salar brines. This study presents the development and characterization of a novel composite membrane adsorbent based on a monoclinic β-Li_2_TiO_3_ (LTO) lithium-ion sieve embedded within a poly­(vinyl chloride) (PVC) and silica (SiO_2_) matrix. The incorporation of SiO_2_ as a structural component enhances mechanical stability, processability, and wettability of the membrane enabling fabrication via industrially scalable extrusion and calendaring techniques and promoting contact of the Li-bearing brine with LTO particles inside the pore system of the membrane. Three membrane compositions with varying SiO_2_:LTO ratios (50:50, 40:60 and 30:70 wt %) were systematically investigated. Among the investigated compositions, the 40% SiO_2_-60% LTO membrane achieved the highest equilibrium adsorption capacity of approximately 37 mg Li^+^ g^–1^ HTO at 45 °C from a dilute feed solution (*C*_0_ = 100 mg L^–1^), consistent with endothermic, chemisorption-controlled Li^+^/H^+^ exchange kinetics best described by the pseudo-second-order model. Selectivity evaluation in a mixed-cation solution simulating geothermal brine composition yielded a Li^+^ distribution coefficient (*K*_d_) of 304 mL g^–1^, with separation factors of 12 over Na^+^ and Mg^2+^, and 8 over K^+^. The membrane retained stable adsorption capacity over several consecutive adsorption–desorption cycles, confirming good chemical and structural stability. These results demonstrate the potential of PVC/SiO_2_/LTO composite membranes as a promising, cost-effective, and industrially scalable platform for selective lithium recovery from complex brines.

## Graphical abstract

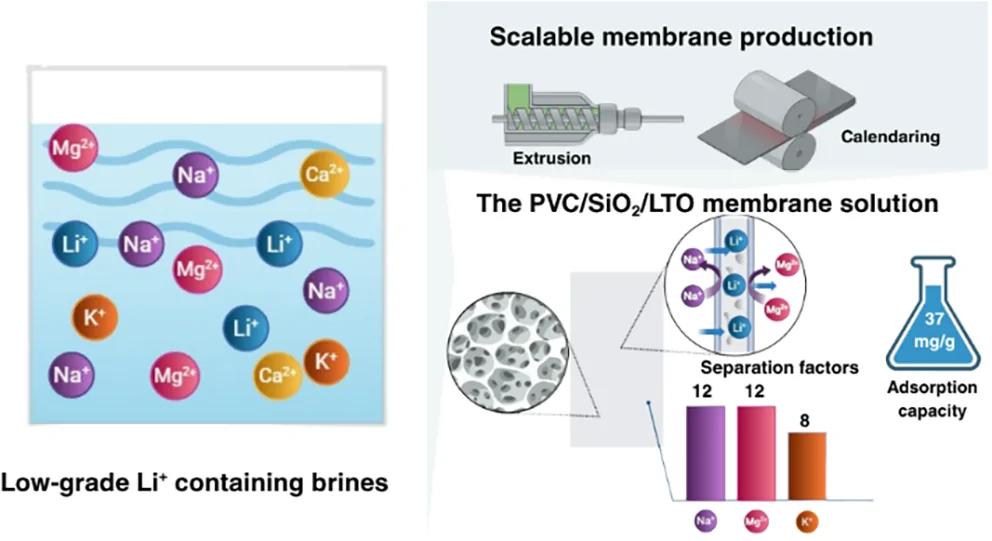

## Introduction

Lithium has become one of the strategically most important mineral resources due to its essential role in energy storage components for portable electronic devices and the rapidly expanding electric vehicle industry.^1–3^ This explosive growth in Li demand suggests a challenge concerning its supply in the future. Although traditionally mined from hard rock ores and continental brine deposits, these resources may not be sufficient to meet the future needs. Consequently, extracting lithium from abundant, low-grade liquid resources, such as salt-lake brines, seawater and geothermal brines, has become an urgent research priority.^4,5^ The challenge inherent in utilizing these liquid sources lies in their complex compositions: Li^+^ concentrations are typically very low, often coexisting with extremely high concentrations of competing ions, particularly Na^+^, K^+^, Ca^2+^, and Mg^2+^. Notably, the radii of Mg^2+^ and Li^+^ are similar (e.g., Mg is 0.072 nm and Li is 0.074 nm), making their separation particularly difficult.^6^

Traditional extraction methods, such as precipitation and solvent extraction, are often energy-intensive, time-consuming, and environmentally unfriendly. Therefore, the use of highly selective lithium-ion sieves (LIS) has emerged as a promising, simple, and effective technique for Li recovery due to its better selectivity, low operating cost, and environmental compatibility.^7–9^

A number of lithium-ion sieves (LIS) have been described in literature, including manganese-based LIS (LMO) and titanium-based LIS (LTO), such as Li_1.6_Mn_1.6_O_4_ and Li_2_TiO_3_ LIS.^10^ Titanium-based LIS are particularly attractive due to their strong Ti–O bond, which provides superior structural stability and excellent acid resistance compared to LMO, resulting in minimal titanium dissolution during regeneration.^5–7^

Historically, most LIS materials have been synthesized in powder form, which poses practical issues in large-scale columns, including poor fluid dynamics, significant pressure drop, and particle loss. To overcome these challenges and enable continuous, industrial operation, a significant focus has been placed on forming LIS powders into macroscopic structures such as granules, foams, or composite membranes.^11–16^ LIS membranes represent a critical area of development, offering advantages like high mechanical properties and a geometry that facilitates close contact between the liquid and the active adsorption sites, enabling easier continuous operation.^11^

Various polymers, including pol­(yvinyl chloride) (PVC), poly­(vinyl alcohol) (PVA), and polyacrylonitrile (PAN), have been utilized as binders to immobilize LISs in membrane forms. For instance, PVC is widely used in composite materials because of its low cost, abundance, and good resistance to acid and alkaline media.^17^ Despite the progress in the LIS technology, several key obstacles hinder its widespread industrial deployment.

While titanium-based LIS possess high intrinsic stability, LMO-type adsorbents widely suffer from manganese dissolution during acid regeneration, which drastically reduces their cyclic stability and lifetime. Furthermore, maintaining high Li selectivity in complex brines containing high concentrations of competitive cations, especially Na^+^ and Mg^2+^, remains a major challenge for all LIS materials.^14,18^ Therefore, titanium-based sieves (H_2_TiO_3_/HTO) have an inherent stability advantage.

Processing LIS powders into membranes often leads to loss of hydrophilicity, poor mechanical stability, and therefore reduced Li^+^ adsorption performance.^19^ Developing cost-effective and scalable membrane manufacturing techniques is critical for industrial viability. Many reported LIS composite materials use complicated synthesis methods or specialized membrane matrices, which cannot be scaled cost-effectively.^8,9^

To address these shortcomings, this study focuses on the development and comprehensive characterization of a novel, stable, and cost-effective Li_2_TiO_3_ (LTO)-based lithium-ion sieve membrane. Our approach yields a lithium-ion sieve membrane, synthesized by mixing LTO, the polymer binder PVC, which is a cost-effective and competitive commercial membrane platform (Amer-Sil Abraca membrane platform), and, as a key ingredient, silica SiO_2_ in order to achieve higher structural stability and easier processability. This synthesis route leverages existing commercial membrane fabrication platforms (extrusion and calendering) to ensure cost-effective and scalable production. The incorporation of the SiO_2_ particles has not previously been reported for LIS membranes and was intended to improve wettability, processability, and structural stability during membrane fabrication.

This study evaluated membrane performance using a low initial lithium concentration of 100 mg L^–1^ to simulate the challenging conditions typical of geothermal brines. The best-performing composite membrane achieved a maximum adsorption capacity of approximately 37 mg g^–1^ HTO at 45 °C and demonstrated superior selectivity in the presence of common ions found in brines (Na^+^, K^+^, Mg^2+^), yielding a Li^+^ distribution coefficient (*K*_d_) of 304 mL g^–1^.

## Experimental Section

### Materials

Poly­(vinyl chloride) (PVC) and silica (SiO_2_, particle size distribution ∼8 μm – ∼33 μm) as were supplied by Amer-Sil, as part of their Abraca membrane platform, and used as received. Lithium titanate (Li_2_TiO_3_, LTO, particle size distribution ∼0.30 μm – ∼100 μm) was provided by Treibacher AG and used as the active adsorption material for membrane fabrication. Hydrochloric acid (HCl, ≥37%, Sigma-Aldrich) was used for the delithiation step to convert LTO into the corresponding lithium–ion sieve H_2_TiO_3_. Sodium carbonate (Na_2_CO_3_, ≥99.5%, Fisher Scientific), sodium hydrogen carbonate (NaHCO_3_, 99.7–100.3%, Fisher Scientific), and lithium chloride (LiCl, >98%, TCI Chemicals) were used to prepare model lithium-containing solutions for adsorption experiments. Magnesium chloride hexahydrate (MgCl_2_ × 6H_2_O, 99%, Fischer Scientific) and potassium chloride (KCl, 99%–100.5%, Alfa Aesar) were used to prepare model solution for selectivity tests. Nitric acid (HNO_3_, TraceSel. 69%, Fisher Scientific) was used for ICP sample preparation. Single element ICP standards, Li (1000 ppm), Na (1000 ppm) and Ti (1000 ppm) from LabKings, Mg (1000 ppm) and K (1000 ppm) from Merck Supelco Certipur were used for the calibration line.

### Membrane Preparation

Defined proportions of poly­(vinyl chloride) (PVC), silica (SiO_2_), and lithium titanate (Li_2_TiO_3_, LTO) were dry-mixed to obtain a homogeneous powder blend. Membranes were prepared with varying LTO contents by systematically adjusting the LTO/SiO_2_ ratio while maintaining the overall formulation approach and particle loading is shown in Table S1. Following homogenization, predetermined amounts of an organic solvent and water were added to form a wet precursor mixture, based on a patented procedure.^20^ The resulting wet powder was processed by extrusion through a flat slit die, followed by calendaring to obtain membrane sheets. Subsequently, the membranes were immersed in sequential hot water baths at 70 and 80 °C to remove the solvent and induce pore formation via phase inversion. The membranes were then dried prior to further use.^21^

A series of membranes with different LTO loadings were produced; from this series, three representative compositions were selected for detailed adsorption–desorption studies in order to evaluate the influence of active phase content on lithium uptake performance and cyclic stability.

### Materials Characterization

Mercury porosimetry was carried out on a PoreMaster analyzer from Quantachrome. Prior to analysis, the sample was weighed (approximately 0.3 g) and transferred to an analysis glass tube. The sample was degassed under reduced pressure reaching 1000 mTorr. After this, the sample was degassed for 5 additional minutes (<50 mTorr). The glass tube was refilled with Hg and transferred to the high-pressure analysis station. The pressure was gradually increased from 20 to 33,000 psi. The pore size distribution was determined using 480 erg/cm^2^ as mercury surface tension and 180° as contact angle. Membrane morphology, including particle structure, surface features, and cross sections, was examined using a Zeiss Sigma HD-VP scanning electron microscope (Zeiss, Germany) equipped with an Everhart-Thornley secondary electron detector. Cross-sectional samples were prepared by embedding membranes in epoxy resin. Prior to imaging, all samples were sputter-coated with Au. The identity of the prepared membranes was confirmed via powder X-ray diffraction analysis. A PANalytical EMPYREAN with a PIXcel3D detector was used to obtain wide-angle powder X-ray diffractograms (WA-PXRD) with Cu Kα radiation generated at a voltage of 45 kV and a tube current of 40 mA. Measurements were performed in the Bragg–Brentano HD reflection geometry. The data was recorded in the continuous mode, employing a step size 2-θ of 0.0394°. Inductively coupled plasma optical emission spectroscopy was performed using an Agilent 5110 instrument. The operation conditions were as follows: read time: 5 s, RF power: 1.2 kW, stabilization time: 8 s, viewing mode: axial, viewing height: 8 mm, nebulizer gas flow: 0,65 L min^–1^, plasma flow: 12 L min^–1^, and auxiliary gas flow: 1.2 L min^–1^. Infrared Spectra were recorded on a Bruker Tensor 27 Hyperion FTIR spectrometer (Bruker, USA) equipped with an ATR diamond unit.

The contact angle of the membranes was measured with a Drop shape analysis system DSA10 Mk2 (Krüss, Germany) with five drops of deionized water per sample.

### Membrane Activation

Membrane activation experiments were performed in triplicates. In a batch experiment, a preweighed lithium-ion sieve membrane was treated with 0.2 mol L^–1^ HCl and shaken at 80 rpm for 24 h at three different temperatures (25, 45, and 60 °C). Following activation, LIS membrane was washed thoroughly with deionized water and dried prior to further use. The concentrations of Li^+^ and Ti^4+^ were measured by ICP-OES.

### Adsorption Experiments

Adsorption experiments were conducted in triplicates at three different temperatures (25, 45, and 60 °C). In a batch adsorption experiment, a preweighed lithium-ion sieve membrane (*m*) was immersed in a lithium-containing solution of known volume (*V*) and initial lithium concentration (*C*_0_). The membrane in the model brine was agitated on a circular shaker at 80 rpm for 24 h to ensure equilibrium.

After the adsorption experiment, the membrane was removed from the solution, and the equilibrium lithium concentration (*C*_e_) was determined using ICP-OES. The equilibrium adsorption capacity (*q*_e_) was calculated according to eq 1 ^6,11^

1 $$q_{e}=\frac{C_{0}-C_{e}}{m}\times V$$

where *C*_0_ and *C*_e_ (mg L^–1^) are the initial and equilibrium lithium concentrations, respectively, *V* (L) is the solution volume, and *m* (g) is the mass of the membrane.

All adsorption experiments were performed at pH 9 using a 50 mM Na_2_CO_3_/NaHCO_3_ buffer system containing LiCl to obtain an initial lithium concentration of 100 mg L^–1^. The solid-to-liquid (S/L) ratio was maintained at 6 g L^–1^.

Adsorption kinetics were evaluated by determining the lithium uptake (*q*_t_, mg g^–1^) at different contact times (*t*). At predetermined time intervals, solution samples were collected, and the lithium concentration (*C*_t_, mg L^–1^) was measured by ICP-OES. The adsorption capacity at time *t* was calculated using eq 2 ^6,11^

2 $$q_{t}=\frac{C_{0}-C_{t}}{m}\times V$$

where *C_t_* (mg L^–1^) is the lithium concentration at time *t*.

### Reusability of Lithium-Ion Sieve Membrane

The reusability of the lithium-ion sieve membrane was evaluated through repeated adsorption–desorption cycles under identical experimental conditions (S/L ratio = 6 g L^–1^, *C*_0_ = 100 mg L^–1^, *T* = 25 and 45 °C, 0.2 mol L^–1^ HCl). After each adsorption step, the membrane was separated from the lithium-containing solution and washed with deionized water. Membrane regeneration was done by desorption with 0.2 mol L^–1^ HCl at 25 and 45 °C for 24 h, which displaced adsorbed Li^+^ by H^+^ through the reversible H^+^/Li^+^ exchange mechanism of the HTO lattice.

Following desorption, the solution was collected and analyzed for lithium and titanium concentrations using ICP-OES. The regenerated membrane was rinsed with ultrapure water and reintroduced into a fresh lithium solution to initiate the next adsorption cycle. This procedure was repeated 5 times to evaluate the cyclic stability and regeneration performance of the membranes.

### Selectivity Test

The adsorption performance of the composite membranes was quantified by the equilibrium adsorption capacity (*q*_e_), and their selectivity toward Li^+^ in the presence of competing cations was evaluated by using the distribution coefficient (*K*_d_) and separation factor (SF). The distribution coefficient represents the ratio between the amount of lithium adsorbed onto the membrane and its equilibrium concentration in the solution. The separation factor was calculated as the ratio of the distribution coefficient of Li^+^ to that of another competing cation (M) present in the feed solution, according to eqs 3 and 4 ^11^

3 $$K_{d}=\frac{q_{e}}{C_{e}}$$

4 $$SF=\frac{K_{d}^{Li}}{K_{d}^{M}}$$

## Results and Discussion

At first, the wettability of the membrane materials was determined, as this is a critical parameter for Li^+^ adsorption. As described in the Experimental Section, the main variation parameter was the ratio of LTO and SiO_2_ in the PVC matrix. While LTO is responsible for the Li^+^ adsorption capacity, SiO_2_ provides a hydrophilic surface and structural stability, therefore, it is relevant to determine the ideal ratio. Extreme values, of <20% LTO and >80% LTO, were not investigated in detail, even though the membranes were prepared for test purposes. This was done because, at low LTO ratios (<20%), the expected Li^+^ capacity is too low, while at too high LTO ratios (>80%), the resulting membranes become extremely brittle and mechanically unstable.

### Contact Angle Measurement

Contact angle measurement is a widely used technique to characterize the wettability of a solid surface by a liquid, in this case water. The angle formed between the tangent to the liquid/vapor interface and the solid/liquid interface at this contact line defines the contact angle (θ).^22^ Surfaces with θ < 90° are considered hydrophilic, while surfaces with θ > 90° are considered as hydrophobic. Time-resolved contact angle measurements determine the dynamic evolution of θ immediately after droplet hits the surface and giving insights concerning surface energy, liquid absorption, and spreading kinetics.^23^ For composite surfaces composed of multiple materials, the resulting wettability reflects a combination of the individual surface energies and structural characteristics of each component, making contact angle analysis an effective tool for seeing how variations in composition are influencing overall surface wettability.

Figure 1 (left to right) shows the evolution of the contact angle over time (2 s) for three membrane compositions: 50% SiO_2_-50% LTO, initial θ = 35.5°, decreasing to 17.9°, 40% SiO_2_-60% LTO, initial θ = 47.5°, decreasing to 32.9° and 30% SiO_2_-70% LTO, initial θ = 59.6°, decreasing to 46.8° after 2 s and showing that all three membrane compositions have hydrophilic nature. The progressive decrease in contact angle with time reflects the spreading and wetting dynamics of water on each membrane surface. Increasing LTO (Figure S1) content is associated with higher initial contact angles, indicating reduced hydrophilicity with higher LTO fraction. These results prove the beneficial effect of SiO_2_ incorporation. In addition to easier processability (pure LTO/PVC mixtures cannot be mixed and calendared efficiently), SiO_2_ incorporation leads to higher hydrophilicity and better wettability.

**Figure 1:**
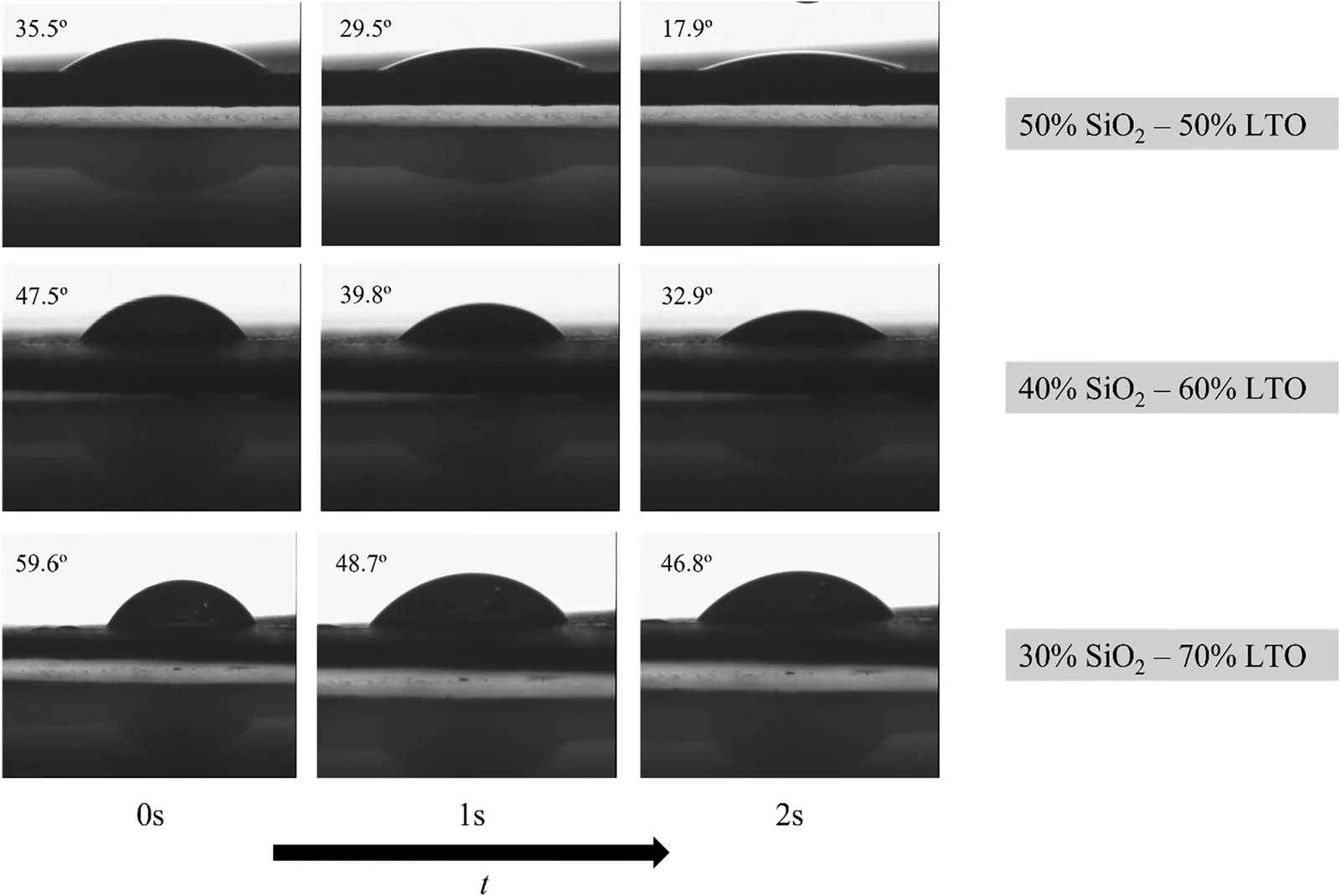
Time-resolved contact angle measurements of water droplets on SiO_2_ - LTO composite surfaces with varying compositions.

### Lithium-Ion Sieve Membrane Characterization

Beside the general wettability, the pore structure of the resulting membranes is a decisive factor for the Li^+^ adsorption performance. The pore structure of the composite membranes was characterized by mercury intrusion porosimetry, and the resulting pore size distributions are presented in Figures 2 and S2. Mercury porosimetry is particularly suitable to determine the accessible pore volume in the membranes, as it determines truly accessible pore volume.^24^ All three membranes exhibited a bimodal pore size distribution, with a broad shoulder in the macropore region (ca. 1–0.5 μm) and a more pronounced peak in the mesopore/small macropore region (ca. 0.1–0.02 μm), which is characteristic of phase-inversion-derived asymmetric membranes.

**Figure 2:**
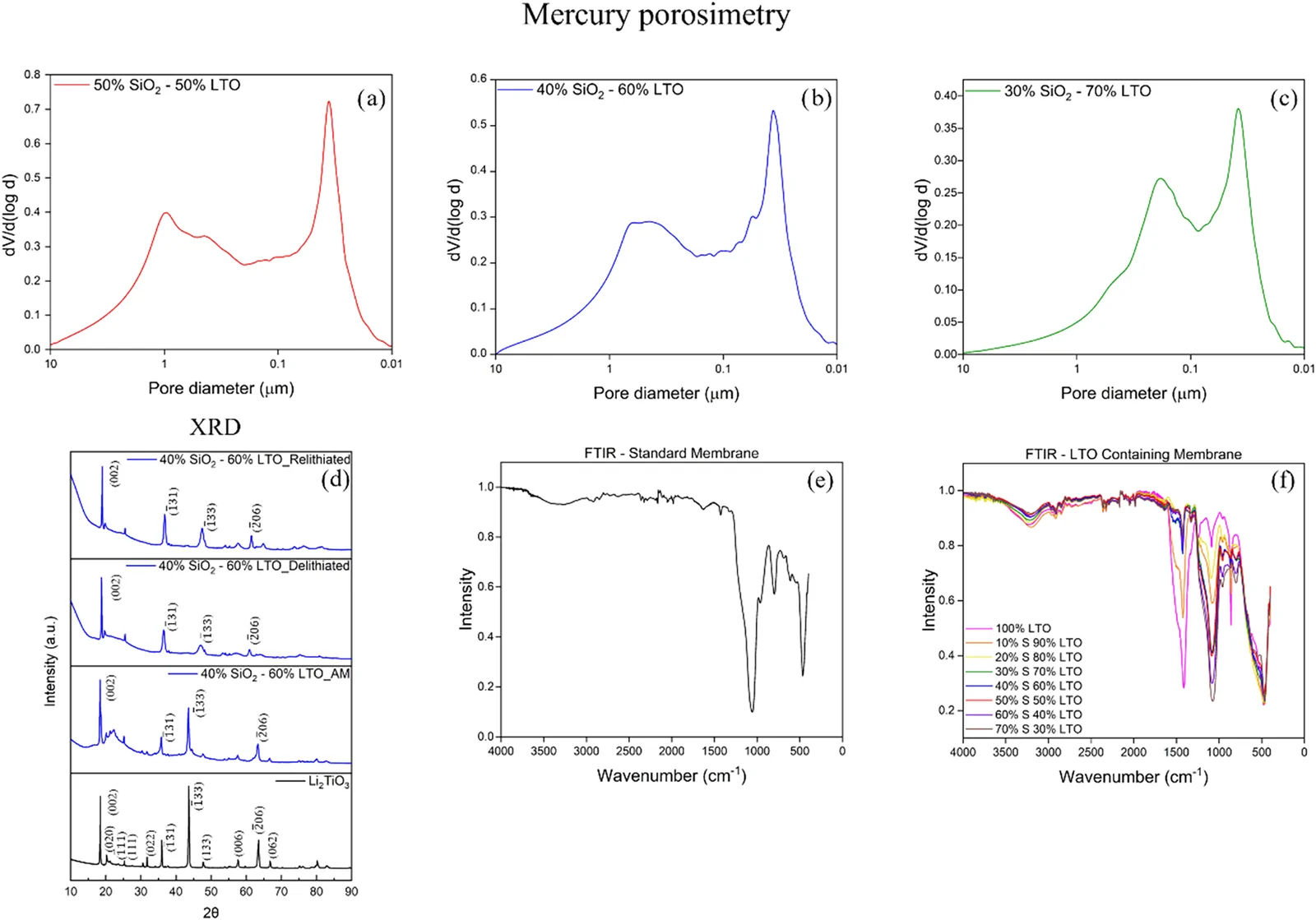
Physicochemical characterization of SiO_2_/Li_2_TiO_3_ (LTO) composite membranes: (A–C) Hg porosimetry pore size distributions for varying SiO_2_:LTO ratios, (D) XRD patterns of 40% SiO_2_-60% LTO in as-made, delithiated, and relithiated states, (E, F) FTIR spectra of standard and LTO containing membranes across different compositions.

The lower diameter maximum is attributed to the voids in between silica and LTO aggregates, while the larger-diameter feature corresponds to macrovoids generated by rapid solvent extraction during membrane casting.^25,26^ As the LTO content increased from 50% to 70%, with a concurrent reduction in SiO_2_ content from 50% to 30%, a systematic decrease in total pore volume (Table S2) was observed, from 0.71 cm^3^ g^–1^ to 0.54 cm^3^ g^–1^ and further to 0.43 cm^3^ g^–1^, respectively. This progressive densification of the membrane structure is attributed to two concurrent effects: a lower amount of SiO_2_, which acts as a particulate porogen in the PVC matrix, and the increased packing of LTO particles, which occupies pore space within the polymer network.^27,28^ The inverse relationship between LTO loading and pore volume indicates that the SiO_2_ content plays a critical role in governing the final membrane porosity, and its systematic substitution with LTO provides a controllable means of tuning the pore structure of the composite membranes. The hierarchical porosity is advantageous for adsorption-driven separations as macropores facilitate adsorbent solution penetration and reduce external mass-transfer resistance, whereas smaller pores enhance internal surface accessibility. However, excessive filler loading may slow the lithium diffusion to internal adsorption sites.^29,30^

Additionally, it is important to determine if the membrane manufacturing, alters the initial LTO structure, therefore XRD analysis was conducted. The XRD analysis (Figure 2d) confirms that LTO exhibits a monoclinic layered structure consistent with β-Li_2_TiO_3_.^6,11,12,31^ The high intensity and narrow reflections indicate good crystallinity of the LTO phase. The XRD pattern of the as-made (AM) composite membrane (40% SiO_2_-60% LTO_AM) retained all major LTO diffraction peaks, demonstrating that the crystalline structure of LTO was preserved during the membrane fabrication process. This was also the case for the other LTO/SiO_2_ ratios as illustrated in Figure S3. The broad background signal at ∼20° and reduced diffraction peak intensities relative to the pure LTO powder are attributed to the amorphous nature of the SiO_2_ and PVC matrix constituents.^32–34^ After activation, slight peak broadening and minor shifts toward higher 2θ values were observed, indicating Li^+^/H^+^ exchange and formation of H_2_TiO_3_ while preserving the layered framework. The diffraction peak shifts toward higher 2θ values reflect slight lattice contraction due to replacement of Li^+^ (0.076 nm) by smaller H^+^ (0.0012 nm) cation.^11^ The XRD pattern after relithiation closely resembles that of the original LTO phase, indicating reversible Li^+^ insertion. However, slight differences in diffraction peak intensity and line shape compared to fresh LTO suggest that structural recovery is not identical as Li^+^ does not always occupy the same crystallographic sites as in fresh LTO. Similar observations have been reported for H_2_TiO_3_ ion-sieve systems, where reinsertion of Li^+^ results in partial reconstruction of the layered structure, while a fraction of lithium remains electrostatically associated with the titanate framework rather than occupying fully ordered crystallographic sites.^6,11^ The presence of the amorphous PVC/SiO_2_ matrix may further restrict complete lattice reorganization. Overall, the XRD analysis demonstrates that the composite membrane architecture consists of well-preserved monoclinic LTO crystallites uniformly embedded within an amorphous PVC/SiO_2_ matrix.

FTIR spectra confirm the coexistence of PVC, silica, and LTO components without formation of new chemical bonds during membrane preparation. Characteristic bands at the range of 3200–3600 cm^–1^ are attributed to the tensile vibration of the OH group in the material. Bands at the range of 2900–3000 cm^–1^ could be attributed to C–H stretching, possibly from PVC. Vibration bands in the range of 1600–1650 cm^–1^ are attributed to H–O–H bending from water adsorbed by hydroxyl groups.^35^ Bands in the range of 1000–1100 cm^–1^ are attributed to Si–O–Si stretching from silica and bands in the range of 600–800 cm^–1^ could be attributed to C–Cl stretching from PVC and possibly Ti–O stretching from LTO.^36–38^ This combined effect could explain why those bands appear to be stronger and broader. Importantly, no significant peak shifts or new absorption bands are observed, indicating that composite formation is dominated by physical embedding rather than chemical interactions between phases. This observation is consistent with the powder XRD results showing preserved crystallinity of LTO and supports the interpretation that structural changes observed in porosity and adsorption behavior arise primarily from morphological evolution rather than chemical modification.

### Scanning Electron Microscopy (SEM)

The surface morphology, cross-sectional structure and EDS mapping of the composite membranes were examined by SEM, and images are presented in Figures 3 and S4–S6. The cross-sectional views (Figure 3a and 3d) of all three membranes reveal an asymmetric structure characteristic of phase inversion derived membranes, consisting of a relatively dense skin layer supported by a more porous sublayer containing macrovoids up to 100 μm. The macrovoids visible in the cross sections are consistent with finger-like pore formation driven by rapid solvent/nonsolvent exchange during the immersion precipitation process.^39,40^

**Figure 3:**
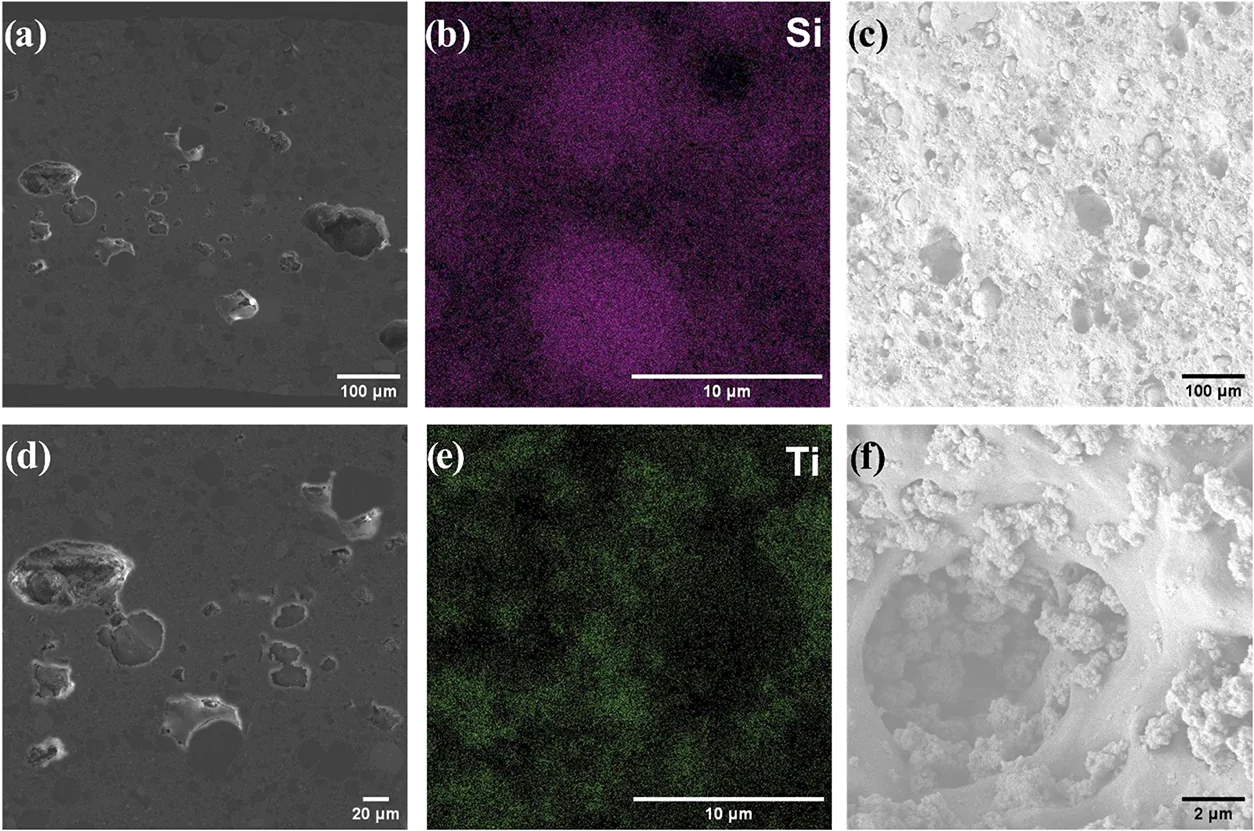
SEM images of the 40% SiO_2_-60% LTO membrane: (a, d) cross-sectional views, (b, e) EDS mapping of Si and Ti, and (c, f) surface views.

The surface views (Figure 3c and 3f) show that LTO particles are present and distributed across the membrane surface, with the higher-magnification images (Figure 3f) revealing the characteristic granular morphology of the LTO agglomerates partially exposed at or near the surface. A notable trend is observed with increasing LTO content: the surface of the 50% LTO membrane (Figure S5) appears relatively smooth with well-defined circular macropores, while the 60% LTO membrane (Figure 3b) exhibits a rougher, more textured surface with denser particle coverage, and the 70% LTO membrane (Figure S6) displays the most compact and least porous surface morphology. This progressive densification of the surface is consistent with the systematic decrease in total pore volume measured by mercury intrusion porosimetry (0.71 → 0.54 → 0.43 cm^3^ g^–1^), and supports the conclusion that higher LTO loading, accompanied by reduced SiO_2_ content results in a denser membrane structure with fewer and smaller surface pores available for ion transport.

The cross-sectional images and EDS mapping further confirm that LTO particles are embedded throughout the bulk of the membrane rather than being confined to the surface, which is important for ensuring that the ion-sieve functionality is distributed across the full membrane thickness and accessible during adsorption–desorption cycling.^41^

### Kinetics of Lithium Adsorption

After characterization of the membrane materials, their capacity for Li^+^ adsorption was determined using standardized solutions.

The adsorption kinetics of Li^+^ onto the composite membranes were investigated at room temperature, 45 and 60 °C for all three membrane compositions (50% LTO, 60% LTO, and 70% LTO), at a comparably low initial Li^+^ concentration of *C*_0_ = 100 mg L^–1^, pH = 9, and a solid to liquid ratio of 6 g L^–1^. The lower Li^+^ concentrations were chosen deliberately, to simulate challenging conditions and Li^+^ concentrations, as they would occur in geothermal brines.^42^ The time-dependent adsorption profiles for all membranes (Figure 4) exhibit a characteristic two-stage behavior: a rapid initial uptake during the first 200–300 min, followed by a gradual approach to equilibrium as the available adsorption sites become progressively occupied. Equilibrium was reached at approximately 1440 min (24 h) for all membrane compositions and temperatures, which is consistent with the relatively slow Li^+^/H^+^ ion-exchange kinetics typical of H_2_TiO_3_-based lithium-ion sieves and the additional mass transfer resistance introduced by the polymeric membrane matrix.^19^

**Figure 4:**
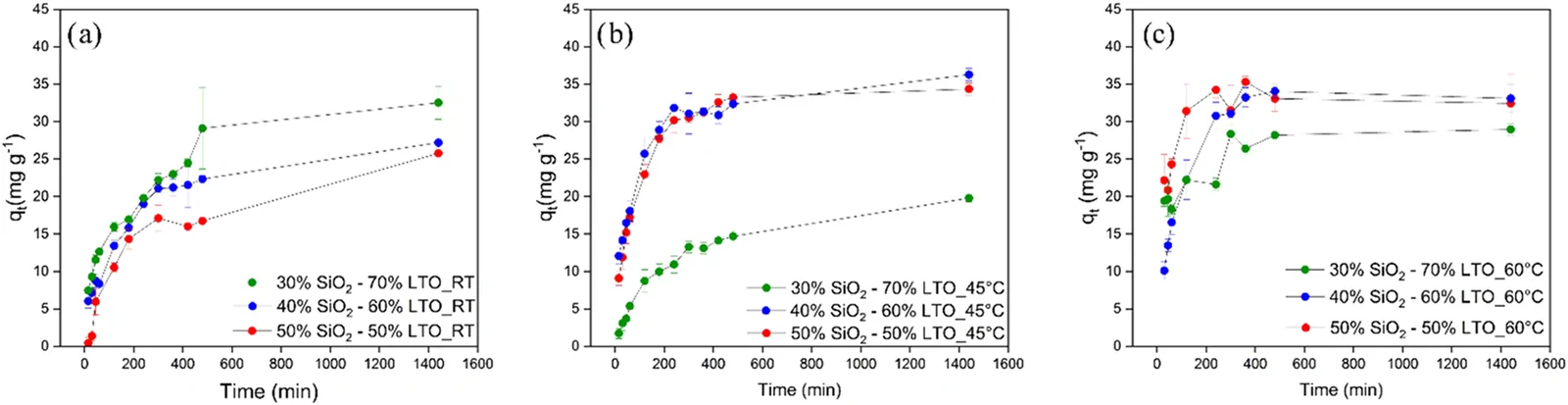
Lithium adsorption kinetics of PVC/SiO_2_/LTO composite membranes (50%, 60%, and 70% LTO loading) at (a) 25 °C, (b) 45 °C, and (c) 60 °C. *C*_0_ = 100 mg L^–1^, pH = 9, S/L ratio = 6 g L^–1^.

A clear positive effect of temperature on adsorption capacity was observed across all membrane compositions. At room temperature, the 40% SiO_2_-60% LTO membrane reached an equilibrium capacity of approximately 25 mg Li^+^ g^–1^ HTO, which increased to ∼37 mg Li^+^ g^–1^ HTO at 45 °C and similarly ∼35 mg g^–1^ at 60 °C, confirming the endothermic nature of the Li^+^ adsorption process.^19^ The enhancement in the lithium adsorption capacity (*q*_e_) with increasing temperature is attributed to the increased mobility of Li^+^ ions in solution, reduced solution viscosity, and enhanced ion diffusion through the membrane pores and into the HTO lattice at elevated temperatures, which is consistent with diffusion-controlled lithium adsorption reported for titanium-based ion sieves.^11^

At 45 °C (Figure 4b), the 50% and 60% LTO membranes exhibit closely overlapping kinetic profiles, indicating that increasing the nominal LTO content from 50% to 60% does not produce a proportionate increase in accessible adsorption capacity under these conditions. At elevated temperature, improved Li^+^ mobility and diffusion facilitate efficient utilization of accessible HTO sites in both membranes, such that the additional LTO in the 60% composite contributes comparatively little extra capacity. This limitation becomes more pronounced for the 70% LTO membrane, which shows lower uptake despite its higher nominal active-phase content, consistent with the mercury-porosimetry results showing progressive membrane densification and reduced pore connectivity with increasing LTO loading.

Interestingly, the 40% SiO_2_-60% LTO membrane shows the most balanced performance, combining relatively high capacity (∼37 mg g^–1^) with favorable kinetic behavior. Although higher LTO loading increases the number of adsorption sites, excessive LTO content may reduce effective pore connectivity, thereby limiting lithium transport pathways. This interpretation is supported by mercury porosimetry results showing reduced total pore volume with increasing LTO fraction, indicating that structural densification may hinder diffusion at higher loadings. Furthermore, LTO would be a significant cost factor in scaled production as industrial-grade/battery grade LTO prices, range from 8.60 to 86 EUR/kg^43^ depending on purity and quantity, whereas industrial-grade SiO_2_ costs only 0.05 to 3.50 EUR/kg^44^ and PVC ranges from 0.6 to 1 EUR/kg,^45^ therefore the optimal material shall contain the lowest amount of LTO possible, to achieve maximum adsorption capacity. A preliminary techno-economic assessment based on these price ranges (Table S3) confirms that Li_2_TiO_3_ dominates the raw material cost of the membrane, accounting for 79–98% of the total despite comprising only 33.5 wt % of its mass.

Concerning the operation temperature, 45 °C is the optimal temperature, as it clearly increases Li^+^ capacity and the maximum capacity is reached quicker. Further increase of the temperature to 60 °C does neither increase the adsorption speed nor capacity significantly, but would require additional energy costs to heat the solution. Moreover, longer use and operations at 60 °C raises concern regarding the long-term stability of the PVC binder, as progressive dechlorination and HCl evolution has been reported to occur at moderate temperatures over longer exposure, which could compromise the mechanical stability of the membrane.^46,47^

Overall, these findings indicate that lithium adsorption in composite membranes is governed by coupled effects of active phase availability, pore architecture, and temperature-dependent mass transfer processes. The highest adsorption capacity achieved in this study (37 mg g^–1^) was obtained at 45 °C temperature, highlighting the importance of structural optimization and operating conditions to balance adsorption capacity and transport kinetics.

To demonstrate the overall rate of adsorption in the LIS composite, pseudo-first (eq 5) and pseudo-second-order (eq 6) equations were used, where *k*1 and *k*2 are their respective order constants.^11,48^

5 $$\log\left(q_{e}-q_{t}\right)=\log\left(q_{e}\right)-\frac{k1}{2.303}\times t$$

6 $$\frac{t}{q_{t}}=\frac{1}{k_{2}xq_{e}^{2}}+\left(\frac{1}{q_{e}}\right)\times t$$

To clarify the rate-controlling mechanism of Li^+^ adsorption, the kinetic data for the 40% SiO_2_-60% LTO membrane were fitted to the Weber-Morris intraparticle diffusion model, pseudo-first-order, and pseudo-second-order kinetic models (Figure 5). The pseudo-second-order model provided a significantly better fit to the experimental data (*r* ^2^ = 0.97) compared with the pseudo-first-order model (*r* ^2^ = 0.92). The superior fit of the pseudo-second-order model indicates that the rate-limiting step of Li^+^ adsorption onto the composite membrane is chemisorption, specifically the Li^+^/H^+^ ion exchange at the active sites of the HTO lattice, rather than solely intraparticle diffusion.^36,49^ The same trend was observed across all membrane compositions, with the pseudo-second-order model consistently providing a better fit (*r* ^2^ = 0.97–0.99) than the pseudo-first-order model (*r* ^2^ = 0.92–0.96) and the pseudo-second-order *q*_e_ values closely matching the experimental equilibrium capacities (Table S4). This is consistent with findings reported for HTO powders and other HTO-based composite adsorbents in the literature, where the pseudo-second-order model has been widely adopted to describe the overall adsorption kinetics.^6,11,50^ This demonstrates that embedding the HTO material into the membrane does not alter the fundamental Li^+^ adsorption mechanism drastically.

**Figure 5:**
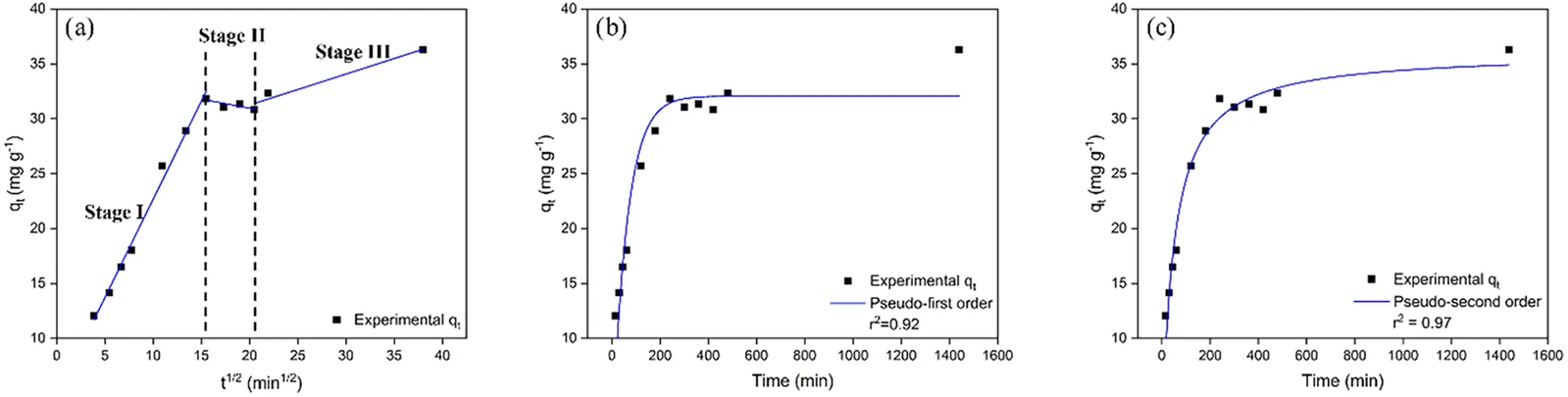
Kinetic models for Li^+^ adsorption by LIS composite: (a) Weber-Morris intraparticle diffusion model, (b) pseudo-first- order and (c) pseudo-second-order reaction rate models at 45 °C.

Expressed in terms of practical Li^+^ removal from solution, the 40% SiO_2_-60% LTO membrane achieved the highest extraction efficiency (73.0% at 24 h) and fastest initial removal rate (1.62 mg L^–1^ min^–1^) among the three compositions tested, outperforming both the 50% SiO_2_-50% LTO (57.6%, 1.02 mg L^–1^ min^–1^) and 30% SiO_2_-70% LTO (46.5%, 0.27 mg L^–1^ min^–1^) membranes (Table S5). A Weber-Morris plot was examined to gain further insights. The plot exhibits a strongly positive initial region followed by a short near-plateau region and a final slower uptake regime. Overall, the multilinear behavior and nonzero intercepts indicate that pore diffusion contributes to Li^+^ adsorption resistance but is not the sole rate-controlling process. The three adsorption stages have been identified in previous literature as (i) liquid film diffusion, in which Li^+^ transport across the boundary layer is limited by hydrodynamic resistance; (ii) intraparticle diffusion, involving Li^+^ penetration into the membrane pores; and (iii) Li^+^-H^+^ ion exchange, which proceeds progressively until equilibrium is reached.^51^

### Recyclability of Lithium-Ion Sieve Membrane

Long-term performance stability is a critical requirement for practical lithium-ion sieve adsorbents, as repeated adsorption–desorption cycling under acidic conditions can lead to structural degradation and loss of active material. To evaluate the suitability of the prepared composite membranes for sustained use, their reusability was assessed over five consecutive adsorption–desorption cycles by monitoring the lithium adsorption capacity (*q*_e_) as a direct measure of performance consistency, and the concentration of leached titanium (Ti^4+^) in the desorption solution as an indicator of chemical stability.^11,49^ The lithium adsorption capacity (*q*_e_) remained stable throughout the cycling experiment at room temperature, with values ranging from approximately 15.5 mg Li^+^ g^–1^ in cycle 1 to 14.7 mg Li^+^ g^–1^ in cycle 5 at room temperature (Figure 6b). This performance consistency demonstrates that the PVC/SiO_2_ matrix effectively immobilizes the LTO particles and prevents significant adsorbent loss during repeated acid–base treatment.

**Figure 6:**
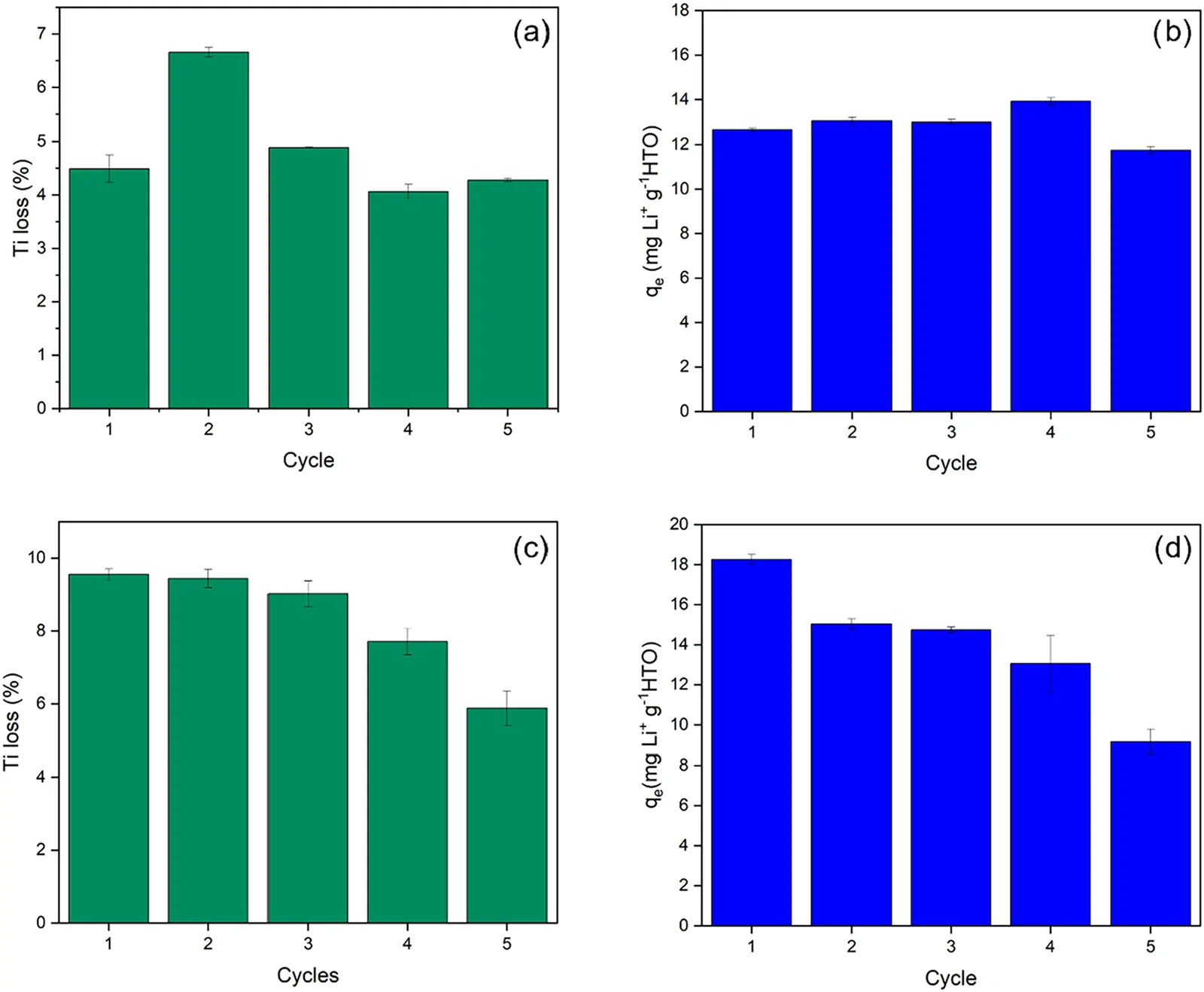
(a) Ti^4+^ loss and (b) Li^+^ adsorption capacity from five adsorption–desorption cycles at room temperature; (c) Ti^4+^ loss and (d) Li^+^ adsorption capacity from five adsorption–desorption cycles at 45 °C.

The chemical stability of the membrane was assessed by quantifying the titanium (Ti^4+^) dissolved into the desorption solution after each cycle (Figure 6a). Ti loss was highest during the second cycle (∼6%), likely attributable to the removal of loosely bound or surface-exposed LTO particles (as shown in Figure S7) during initial acid treatment, after which the dissolution rate decreased and stabilized in the range of 3.5–5% for subsequent cycles. While this Ti^4+^ loss is somewhat higher than values typically reported for pure HTO powders, it remains within the similar range than reported before for polymeric composite membranes. Furthermore, the Li^+^ adsorption capacity remains similar and does not show a statistically significant reduction over various cycles, indicating that the moderate Ti^4+^ leaching does not affect the functionality of the membrane. The structural integrity of the membrane adsorbent was further confirmed by XRD analysis of the membranes after the first and fifth desorption cycles (Figure S8). Both postcycling diffraction patterns closely resemble that of the as-made membrane, retaining the characteristic peaks of the monoclinic Li_2_TiO_3_ phase, with no emergence of new crystalline phases or evidence of structural collapse.

To further investigate the mechanism underlying Ti^4+^ loss, SEM cross-sectional imaging and EDS elemental mapping (Si, Ti) were performed on the as-made, delithiated, and relithiated membranes (Figures S9–S11). Cross-sectional SEM images reveal a progressive increase in surface porosity and void formation from the as-made to the delithiated and relithiated states, consistent with the physical detachment of loosely bound LTO particles from the PVC/SiO_2_ matrix rather than uniform dissolution of the Ti–O framework. EDS mapping confirms that Ti and Si remain colocalized within discrete particulate domains throughout cycling, indicating that the remaining LTO particles retain their structural integrity and that Ti loss occurs predominantly through whole-particle release rather than gradual leaching of the crystal lattice.

On the other hand, cycling stability done at 45 °C is showing a different trend. Ti^4+^ dissolution is higher overall (9–10% in first cycles vs 6% at room temperature). The combined effect of elevated temperature and physical particle detachment accelerates acid-driven dissolution of the Ti–O framework during desorption. Li^+^ adsorption capacity drops steeply across cycles compared to room temperature and this is a direct consequence of greater structural degradation from accelerated Ti^4+^ dissolution at higher temperature, leaving fewer active adsorption sites available with each cycle. Collectively, these results indicate that for long-term operation, room-temperature conditions are preferred, where the material demonstrates good cyclability and chemical stability. Nevertheless, the future work should disentangle the relative contributions of chemical dissolution and physical particle detachment to the observed Ti^4+^ losses, and focus on membrane optimization strategies to minimize these losses, especially during the initial cycling stage as a requirement for deployment in extended operations.

### Selectivity

To determine the suitability of the membranes for realistic applications, the Li^+^ selectivity is a decisive parameter, as in practical application, Li^+^ will be present in a mixture with many other cations, depending on the exact nature of the feedstock.

The selectivity of the 40% SiO_2_-60% LTO composite membrane toward Li^+^ in the presence of competing cations was evaluated using a mixed ion solution containing Li^+^, Na^+^, Mg^2+^, and K^+^. The concentrations of the other cations are listed in Table 1, to resemble the composition of geothermal brines^42,52,53^ where Na^+^ dominates, followed by Mg^2+^ and K^+^, while Li^+^ is always a trace component. No turbidity or precipitate formation was observed in the simulated brine solutions throughout the adsorption experiments, indicating that all cation salts remained fully dissolved under the tested conditions. The distribution coefficient (*K*_d_) of Li^+^ was 304 mL g^–1^, substantially higher than those of Na^+^ (24 mL g^–1^), Mg^2+^ (25 mL g^–1^), and K^+^ (37 mL g^–1^), demonstrating the strong preferential affinity of the membrane adsorbent for lithium ions. Separation factor (SF) greater than 1 indicates preferential adsorption of Li^+^ over the competing ion, and higher values correspond to greater selectivity. Thus, SF values of 12 for Li^+^/Na^+^ and Li^+^/Mg^2+^, and 8 for Li^+^/K^+^, indicate that the membrane adsorbs Li^+^ significantly more favorably than these competing cations, even when present at much higher concentrations in the feed solution. The high selectivity toward Li^+^ is attributed to the ion-sieving mechanism of the H_2_TiO_3_ lattice, wherein the specific cavity size of the delithiated structure preferentially accommodates Li^+^ (ionic radius 0.076 nm) over the larger Na^+^ (0.102 nm), and K^+^ (0.138 nm). Although Mg^2+^ (0.072 nm) has a comparable ionic radius to Li^+^, its significantly higher dehydration energy barrier effectively limits its penetration into the HTO lattice, resulting in similarly low uptake.^11^ These results demonstrate that the PVC/SiO_2_/LTO composite membrane retains the intrinsic ion selectivity of the HTO ion sieve, confirming that the membrane fabrication process does not compromise the selectivity performance of the embedded adsorbent.

**Table 1:** Distribution Coefficients (*K*_d_) and Separation Factors (SF) of the 40% SiO_2_-60% LTO Composite Membrane for Competing Cations

cations	*C*_0_ (mg L^–1^)	*K*_d_ (mLg^–1^)	SF
Li^+^	100	304	1
Na^+^	1051	24	12
Mg^2+^	140	25	12
K^+^	58	37	8

The distribution coefficients (*K*_d_) and separation factors (SF) for the 40% SiO_2_-60% LTO composite membrane (Table 1) demonstrate that the intrinsic ion-sieve effect of the LTO is successfully preserved within the PVC/SiO_2_ matrix. The *K*_d_ for Li^+^ (304 mL g^–1^) is comparable to or even exceeds values reported for other macro-form titanium-based composites, such as HTO/PVA foams or HTO-PVB granules, which also show high selectivity for lithium over competing alkali and alkaline earth metals.^12,18,54^ While the SF_Li/Mg_ and SF_Li/Na_ of 12 are lower than the extremely high separation factors (often >5000) reported for unsupported HTO powders, such a reduction is recognized in composite materials where the polymer binder can partially block active sites or create mass transfer resistance.^55^ Importantly, the present membrane achieves these results under a low initial lithium concentration (100 mg L^–1^), whereas many literature benchmarks for pure powders are established using much higher concentrations (e.g., 669–7000 mg L^–1^) that provide a higher driving force for adsorption and can be seen in Table S6.^4,56^ Consequently, the ability of this scalable PVC/SiO_2_ platform to maintain selective lithium capture in the presence of significant concentrations of Na^+^ (1051 mg L^–1^) and Mg^2+^ (140 mg L^–1^) highlights its practical viability compared to previous conventional PVC membranes that experienced higher capacity losses.^1^ It should be noted that the present SF values reflect preferential Li^+^ enrichment under single-stage equilibrium conditions and are not, by themselves, sufficient to yield battery-grade lithium products, as with other adsorption-based lithium recovery processes, direct production of battery-grade Li_2_CO_3_ or LiOH would require subsequent staged elution and downstream purification steps, positioning the membrane as a lithium capture and enrichment unit within a broader process chain rather than a stand-alone production route.^57^

## Conclusion

The development of an LTO-based lithium-ion sieve (LIS) membrane incorporating SiO_2_ as a structural additive provides a scalable platform for selective lithium recovery from complex brines.

This research provides a novel approach to high-performance LIS membranes by successfully transitioning LIS materials from problematic powder forms into macroscopic composite membranes and demonstrates a balanced lithium uptake capacity and kinetics in a hierarchically porous structure. An important aspect of this work is the incorporation of SiO_2_ particles into a PVC matrix. The incorporation of SiO_2_ improved the wettability and facilitated membrane fabrication at higher LTO loadings while maintaining the structural integrity of the composite membrane.

The study advances the field by demonstrating scalable production possibilities through the use of existing commercial membrane fabrication platforms, such as extrusion and calendaring. The use of commercial extrusion and calendaring processes highlights the potential scalability of the membrane fabrication approach, while the adsorption capacity and kinetics results provide an appealing perspective for the enhanced functionality of the resulting membranes. Postsorption SEM-EDX analysis indicated that Ti loss is primarily caused by the detachment of loosely bound, intact LTO particles from the PVC matrix, pointing to insufficient interfacial adhesion as the main degradation mechanism rather than chemical dissolution of the LTO lattice. Strengthening the LTO-PVC interaction is therefore a key target for future work, and could be achieved through surface modification of LTO particles (e.g., functionalization with silanes or hydroxylation), as well as tuning LTO particle size and surface roughness to improve mechanical anchoring within the polymer matrix. Regarding selectivity, the membranes retained a *K*_d_ value sufficient for effective and preferential Li^+^ extraction from simulated brines, although lower than that of pure LTO powder. This reduction is likely attributable to nonselective ion adsorption within the porous SiO_2_/PVC structure itself, rather than a loss of intrinsic LTO selectivity. The 40% SiO_2_-60% LTO membrane composition achieved a notable equilibrium capacity of approximately 37 mg Li^+^ g^–1^ HTO at 45 °C, benefiting from an endothermic adsorption process that enhances ion mobility at elevated temperatures. The membrane retained its intrinsic Li ion-sieving effect and exhibited a distribution coefficient *K*_d_ (304 mL g^–1^) that was significantly lower for competing cations. Sorption performance remained consistent over consecutive cycles, with only minor fluctuations in capacity confirming the structural stability of LTO particles.

In conclusion, this study demonstrates that the PVC/SiO_2_/LTO composite membrane can serve as a highly active titanium-based lithium-ion sieve while enabling fabrication through scalable membrane manufacturing processes. The incorporation of SiO_2_ improved the membrane wettability and facilitated membrane fabrication, supporting the further development of membrane-based lithium-ion sieve materials for lithium recovery from complex brines.

## Supplementary Material



## Notes

Mass composition of PVC, SiO_2_, and LTO for each membrane formulation; contact angle measurements of a series of LTO/PVC/SiO_2_ membrane composites; Hg porosimetry of membrane composites and their pore volume; PXRD and SEM characterization of membrane composites; preliminary techno-economic assessment of membrane composites; kinetic parameters for Li^+^ adsorption, Li^+^ extraction efficiency, and initial removal rate; and comparison of lithium adsorption capacities of this work with values reported in the literature (PDF)

## References

[1] Chung W. J., Torrejos R. E. C., Park M. J., Vivas E. L., Limjuco L. A., Lawagon C. P., Parohinog K. J., Lee S. P., Shon H. K., Kim H., Nisola G. M. (2017). Continuous Lithium Mining from Aqueous Resources by
an Adsorbent Filter with a 3D Polymeric Nanofiber Network Infused
with Ion Sieves. Chem. Eng. J..

[2] Lu B., Wang X., Zhang Y., Wang Q., Jiang F., Lin W., Liu L., Ye P., Lu W., Lu Z., Xu Z. (2025). Beyond Ion Exchange:
Unraveling the Complexity of Lithium-Ion Adsorption
on H2TiO3. ACS Appl. Mater. Interfaces.

[3] Zhang H., Du W., Zhang P., Guo Z. Y., Wang L., Wang J., Ji Z. Y. (2026). Low-Cost, Scalable Integration of LiMn2O4 with Graphene for Selective
Lithium Extraction via Hybrid Capacitive Deionization. Desalination.

[4] Gu D., Sun W., Han G., Cui Q., Wang H. (2018). Lithium Ion
Sieve Synthesized
via an Improved Solid State Method and Adsorption Performance for
West Taijinar Salt Lake Brine. Chem. Eng. J..

[5] Shi X., Zhou D., Zhang Z., Yu L., Xu H., Chen B., Yang X. (2011). Synthesis and Properties
of Li1.6Mn1.6O4
and Its Adsorption Application. Hydrometallurgy.

[6] Chitrakar R., Makita Y., Ooi K. (2014). Lithium Recovery from
Salt Lake Brine by H2TiO3. Dalton Trans..

[7] Zhao B., Qian Z., Guo M., Wu Z., Liu Z. (2021). The Performance
and Mechanism of Recovering Lithium on H4Ti5O12 Adsorbents Influenced
by (110) and (111) Facets Exposed. Chem. Eng.
J..

[8] Chen C. W., Chen P. A., Wei C. J., Huang H. L., Jou C. J., Wei Y. L., Wang H. P. (2017). Lithium
Recovery with LiTi2O4 Ion-Sieves. Mar. Pollut.
Bull..

[9] Chen H. (2025). First-Principles
Predictions of Lithium Adsorption in Doped Titanium Oxide Ion Sieves. ACS Appl. Mater. Interfaces.

[10] Ge S., Wang C., Liu T., Zhen A., Liao K. (2026). Lithium-Ion
Sieves for Selective Lithium Adsorption: A Review of Modifications,
Shaping Strategies, and Mechanisms. ACS Appl.
Mater. Interfaces.

[11] Lawagon C. P., Nisola G. M., Mun J., Tron A., Torrejos R. E. C., Seo J. G., Kim H., Chung W. J. (2016). Adsorptive Li+ Mining
from Liquid Resources by H2TiO3: Equilibrium, Kinetics, Thermodynamics,
and Mechanisms. J. Ind. Eng. Chem..

[12] Zhang Y., Liu J., Yang Y., Lin S., Li P. (2021). Preparation of Granular
Titanium-Type Lithium-Ion Sieves and Recyclability Assessment for
Lithium Recovery from Brines with Different PH Value. Sep. Purif. Technol..

[13] Zhu G., Wang P., Qi P., Gao C. (2014). Adsorption and Desorption
Properties of Li+ on PVC-H1.6Mn1.6O4 Lithium Ion-Sieve Membrane. Chem. Eng. J..

[14] Wang S., Zheng S., Wang Z., Cui W., Zhang H., Yang L., Zhang Y., Li P. (2018). Superior Lithium
Adsorption
and Required Magnetic Separation Behavior of Iron-Doped Lithium Ion-Sieves. Chem. Eng. J..

[15] Gao R., Xin L., Zhang T. (2026). Efficient Lithium Extraction
Using 2D Montmorillonite
Membranes with Robust Nanochannels via a Cation–Organic Interlayer
Interlocking Strategy. Nano Res..

[16] Guo L. P., Guo Z. Y., Wang J., Zhang P. P., Huang Z. H., Ji Z. Y. (2024). Flexible
Lithium Selective Composite Membrane for Direct Lithium
Extraction from High Na/Li Ratio Brine. J. Membr.
Sci..

[17] Lawagon C. P., Nisola G. M., Cuevas R. A. I., Kim H., Lee S. P., Chung W. J. (2019). Development of High
Capacity Li+ Adsorbents from H2TiO3/Polymer
Nanofiber Composites: Systematic Polymer Screening, Characterization
and Evaluation. J. Ind. Eng. Chem..

[18] Limjuco L. A., Nisola G. M., Lawagon C. P., Lee S. P., Seo J. G., Kim H., Chung W. J. (2016). H2TiO3
Composite Adsorbent Foam for Efficient and Continuous
Recovery of Li+ from Liquid Resources. Colloids
Surf., A.

[19] Wang Q., Yang X., Li C., Zhao C., Huang Y., Qiu S., Qin Y., Shi C. (2024). Titanium-Based Lithium Ion Sieve
Adsorbent H2TiO3 with Enhanced Li+ Adsorption Properties by Magnetic
Fe Doping. Sep. Purif. Technol..

[20] Carole, L. Porous Membrane and Method Of Production Thereof. U.S. Patent, US10,561,992B2, 2020.

[21] Lambert, U. Separator For Secondary Electrical Accumulators With Gas Recombination. U.S. Patent, US8,039,140B2, 2011.

[22] Ismail, A. F. ; Khulbe, K. C. ; Matsuura, T. RO Membrane Characterization. In Reverse Osmosis; Elsevier, 2019; Chapter 3, pp 57–90 10.1016/b978-0-12-811468-1.00003-7.

[23] Pan Y., Zhang Y., Thompson C., Liu G., Zhang W. (2024). Electrospun
Lithium Porous Nanosorbent Fibers for Enhanced Lithium Adsorption
and Sustainable Applications. ACS Appl. Mater.
Interfaces.

[24] Egger C. C., Du Fresne C., Raman V. I., Schädler V., Frechen T., Roth S. V., Müller-Buschbaum P. (2008). Characterization
of Highly Porous Polymeric Materials with Pore Diameters Larger than
100 Nm by Mercury Porosimetry and X-Ray Scattering Methods. Langmuir.

[25] Marbelia L., Bilad M. R., Bertels N., Laine C., Vankelecom I. F. J. (2016). Ribbed
PVC-Silica Mixed Matrix Membranes for Membrane Bioreactors. J. Membr. Sci..

[26] Bilad M. R., Marbelia L., Laine C., Vankelecom I. F. J. (2015). A PVC-Silica
Mixed-Matrix Membrane (MMM) as Novel Type of Membrane Bioreactor (MBR)
Membrane. J. Membr. Sci..

[27] Fernandes C. S., Md Nordin N. A. H., Bilad M. R., Matsuura T., Putra Z. A., Wirzal M. D. H., Jaafar J. (2021). Explication of Hydrophobic Silica
as Effective Pore Former for Membrane Fabrication. Appl. Surf. Sci. Adv..

[28] Mohamednour A. E. E., Nordin N. A. H. M., Bilad M. R., Shafie S. N. A., Hizam S. M., Nawi N. I. M. (2023). Quantifying the Impact of Silica
Hydrophilicity and Loading on Membrane Surface Properties through
Response Surface Methodology. J. Membr. Sci..

[29] Xu S., Zheng K., Boruntea C.-R., Cheng D., Chen F., Ye G., Zhou X., Coppens M.-O. (2023). Surface Barriers to Mass Transfer
in Nanoporous Materials for Catalysis and Separations. Chem. Soc. Rev..

[30] Kim S., Lee J. (2020). Spinodal Decomposition:
A New Approach to Hierarchically Porous Inorganic
Materials for Energy Storage. Natl. Sci. Rev..

[31] Ji Z. Y., Yang F. J., Zhao Y. Y., Liu J., Wang N., Yuan J. S. (2017). Preparation of Titanium-Base Lithium
Ionic Sieve with
Sodium Persulfate as Eluent and Its Performance. Chem. Eng. J..

[32] Nallathambi G., Ramachandran T., Rajendran V., Palanivelu R. (2011). Effect of
Silica Nanoparticles and BTCA on Physical Properties of Cotton Fabrics. Mater. Res..

[33] Maddalena R., Hall C., Hamilton A. (2019). Effect of
Silica Particle Size on
the Formation of Calcium Silicate Hydrate [C-S-H] Using Thermal Analysis. Thermochim. Acta.

[34] Al-Muntaser A. A., Abdelghany A. M., Abdelrazek E. M., Elshahawy A. G. (2020). Enhancement
of Optical and Electrical Properties of PVC/PMMA Blend Films Doped
with Li4Ti5O12 Nanoparticles. J. Mater. Res.
Technol..

[35] Marthi R., Asgar H., Gadikota G., Smith Y. R. (2021). On the
Structure
and Lithium Adsorption Mechanism of Layered H2TiO3. ACS Appl. Mater. Interfaces.

[36] Liu J., Xu N. C., Shi D. D., Cao Y., Zhang T. Y., Zhong K. P., Zhao Q., Bian S. J. (2024). Preparation,
Characterization,
and Adsorption Performance of H2TiO3 Lithium Ion Sieves with Homogeneous
Nanoparticles: Kinetics, Thermodynamics, and Mechanism Analysis. ChemistrySelect.

[37] Panwar K., Jassal M., Agrawal A. K. (2015). In Situ Synthesis of Ag-SiO2Janus
Particles with Epoxy Functionality for Textile Applications. Particuology.

[38] Ramesh S., Leen K. H., Kumutha K., Arof A. K. (2007). FTIR Studies
of
PVC/PMMA Blend Based Polymer Electrolytes. Spectrochim.
Acta, Part A.

[39] Chen R., Mao L., Matindi C. N., Liu G., He J., Cui Z., Ma X., Fang K., Wu B., Mamba B. B., Li J. (2022). Tailoring
the Micro-Structure of PVC/SMA-g-PEG Blend Ultrafiltration Membrane
with Simultaneously Enhanced Hydrophilicity and Toughness by in Situ
Reaction-Controlled Phase Inversion. J. Membr.
Sci..

[40] Zhou J., Ren J., Lin L., Deng M. (2008). Morphology Evolution of Thickness-Gradient
Membranes Prepared by Wet Phase-Inversion Process. Sep. Purif. Technol..

[41] Chen Z., Du J., Shi J. (2025). Mxene-Based
Lithium-Ion Sieve Polymer Membrane for
Sustainable Lithium Adsorption. Sep. Purif.
Technol..

[42] Zegeye R. B., Tufa R. A., Santoro S., Inzillo B. M., Aquino M., Politano G. G., Embaye A. S., Argurio P., De Bartolo L., Curcio E. (2025). Sustainable Lithium Recovery from Geothermal Brine *via* Integrated Membrane Distillation–Selective Electrodialysis. Green Chem..

[43] Battery Grade Powder Li2tio3 With High Purity Lithium Titanate.

[44] imarc . Silica Prices, Trend, Chart, Demand, Market Analysis, News, Historical and Forecast Data Report 2026 Edition.

[45] imarc . Poly Vinyl Chloride Prices, Trend, Chart, Demand, Market Analysis, News, Historical and Forecast Data Report 2026 Edition.

[46] Ouazzou H. A., Nedjar M., Hocine S., Belhocine R., Belabbas F. (2022). Investigation of Hydrothermal Aging on Polyvinyl Chloride
(PVC) Used in Medium Voltage Cables. Ann. Chim.:
Sci. Mater..

[47] Ito M., Nagai K. (2007). Analysis of Degradation Mechanism of Plasticized PVC under Artificial
Aging Conditions. Polym. Degrad. Stab..

[48] Ho Y. S., McKay G. (1998). A Comparison of Chemisorption Kinetic Models Applied to Pollutant
Removal on Various Sorbents. Process Saf. Environ.
Prot..

[49] Du J., Ma J., Cui X., Liu X., Wang D., Wang B., Zhang X., Zhai R., Zhu J., Zuo H., Li Q., He X. (2026). Zr and F Co-Modified Li2TiO3 with Obviously Enhanced
Li+ Adsorption Performances. Sep. Purif. Technol..

[50] Li Y., Yang Z., Ma P. (2023). Research Progress
on New Types of
H2TiO3 Lithium-Ion Sieves: A Review. Metals.

[51] Xue F., Shen C., Qian J., Yang X., Ju S. (2026). Mg2+-Mediated
Interlayer Engineering of Li2TiO3@rGO Ion Sieves via Thermal Reduction
for Efficiency Lithium Extraction. Chem. Eng.
Sci..

[52] Liu C., Lowenstein T. K., Wang A., Zheng C., Yu J. (2023). Brine: Genesis
and Sustainable Resource Recovery Worldwide. Annu. Rev. Environ. Resour..

[53] Subasinghe H. C. S., Zhang H., Shi F., Rezaee M., Taleghani A. D. (2025). Critical
Minerals Extraction from Geothermal Brines. Joule.

[54] Wang L., Wang L., Li L. (2023). Preparation of PVC-LMZO Membrane
and Its Lithium Adsorption Performance from Brine. Desalination.

[55] Gao Y., Sun Y., Chen L., Tan Y., Fan R., Hai C., Zhang H. (2025). Progress of Research
in Titanium-Based Lithium-Ion Sieves for the
Extraction of Lithium from Salt Lake Brine. ACS Omega.

[56] Wang S., Zhang M., Zhang Y., Zhang Y., Qiao S., Zheng S. (2019). High Adsorption Performance of the
Mo-Doped Titanium Oxide Sieve
for Lithium Ions. Hydrometallurgy.

[57] Kong L., Yan G., Hu K., Yu Y., Conte N., Mckenzie K. R., Wagner M. J., Boyes S. G., Chen H., Liu C., Liu X. (2025). Electro-Driven Direct
Lithium Extraction from Geothermal Brines to
Generate Battery-Grade Lithium Hydroxide. Nat.
Commun..

