# Supplementary material for: Scalable Membrane-Based Lithium-Ion Sieve Sorbent for Selective Lithium Extraction from Brines
Source: Langmuir. 2026 Oct 4;42(41):30788–99. doi: 10.1021/acs.langmuir.6c04005 (PMC13689906; doi:10.1021/acs.langmuir.6c04005)
Supplement: Supplementary file 1 [file la6c04005_si_001.pdf]

SUPPLEMENTARY INFORMATION

Scalable Membrane-Based Lithium-Ion Sieve Sorbent for Selective Lithium Extraction from Brines

*Ana Ivanković[1,2,3], Adrien Comès[4], Martin Stockhausen[5], Justyna Florek[2], Heinz Ragossnig[6], Cana Khalouche[4], Thilo Hofmann[5], Freddy Kleitz[2] and Christian M. Pichler[1]**

[1]Vienna University of Technology, Institute of Applied Physics, Wiedner Hauptstrasse 8-10, 1040 Vienna, Austria

[2]University of Vienna, Department of Functional Materials and Catalysis, Währinger Strasse 42, 1090 Vienna, Austria

[3]University of Vienna, Vienna Doctoral School in Chemistry (DoSChem), Währinger Strasse 42, 1090 Vienna, Austria

[4]Amer-Sil S.A., 61 Rue d'Olm, L-8281 Kehlen, Luxembourg

[5]University of Vienna, Department of Environmental Geosciences, Josef-Holaubek-Platz (UZA II), 1090 Vienna, Austria

[6]Treibacher Industrie AG, Auer-von-Welsbach-Strasse 1, 9330 Althofen

**Corresponding author email: pichlerc@iap.tuwien.ac.at*

DOCUMENT INFORMATION:

- Number of pages: 21
- Number of figures: 11
- Number of tables: 6

TABLE OF CONTENTS:

S1. Mass composition of membrane composites

S2. Contact angle measurement

S3. Mercury porosimetry

S4. Powder X-ray diffraction of the standard membrane composite and membranes with varying LTO ratios

S5. Scanning electron microscopy (SEM) and powder X-ray diffraction of the standard membrane composite, its delithiated and relithiated forms

S6. Preliminary techno-economic assessment

S7. Kinetic parameters and Li^+ extraction efficiencies of three membrane composites

S1. Mass composition of membrane composites

Table S1. Mass composition of PVC, SiO_2 and LTO for each membrane fomulation.

Name	PVC (g)	SiO_2 (g)	LTO (g)
**Standard Formulation**	20.80	26.35	x
**70% SiO_2 – 30% LTO**	20.80	18.45	7.91
**60% SiO_2 – 40% LTO**	20.80	15.81	10.54
**50% SiO_2 – 50% LTO**	20.80	13.18	13.18
**40% SiO_2 – 60% LTO**	20.80	10.54	15.81
**30% SiO_2 – 70% LTO**	20.80	7.91	18.45
**20% SiO_2 – 80% LTO**	20.80	5.27	21.08
**10% SiO_2 – 90% LTO**	20.80	2.64	23.72
**100% LTO**	20.80	x	26.35

S2. Contact angle measurement

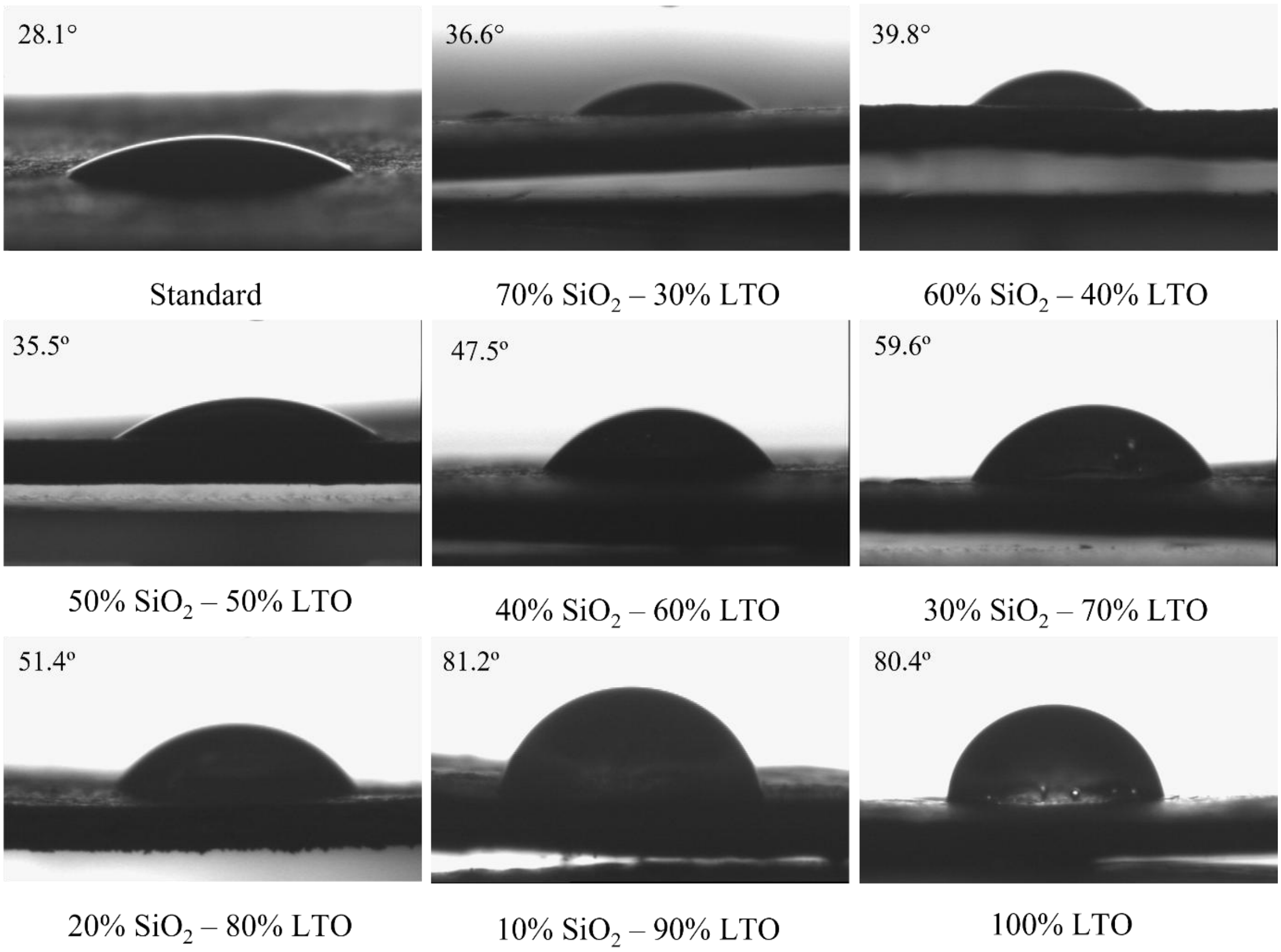

Figure S1. Contact angle measurements of series of membranes with varying LTO ratios.

S3. Mercury porosimetry

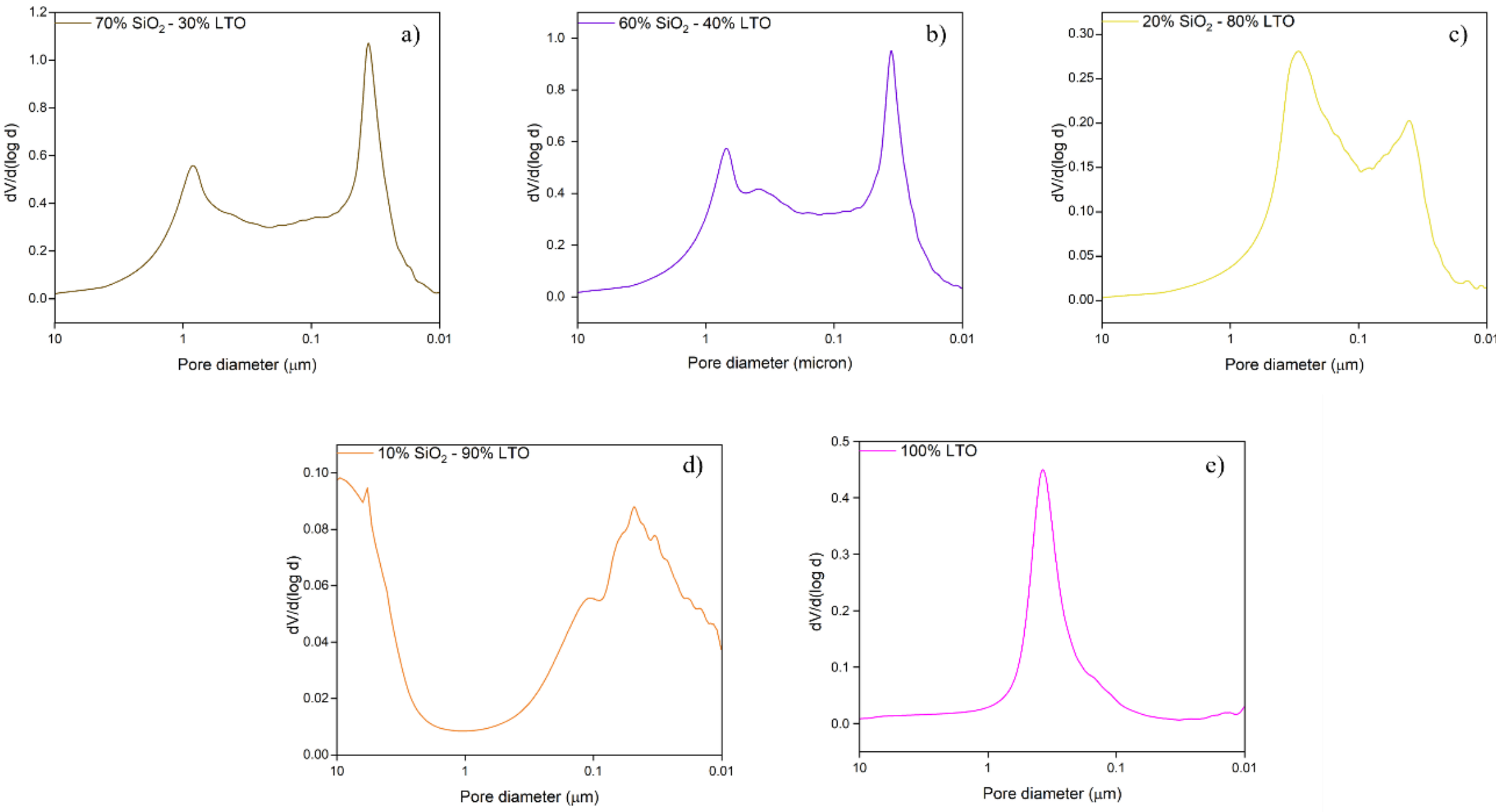

Figure S2. Hg porosimetry pore size distributions for varying SiO_2:LTO ratios, a) 70% SiO_2–30% LTO; (b) 60% SiO_2–40% LTO; c) 20% SiO_2–80% LTO; (d) 10% SiO_2–90% LTO; and (e) 100% LTO.

Table S2. LTO content (%) and corresponding pore volume of composite membranes.

LTO (%)	Pore Volume (cc/g)
**30**	0.86
**40**	0.82
**50**	0.71
**60**	0.54
**70**	0.43
**80**	0.29
**90**	0.15
**100**	0.19

S4. Powder X-ray diffraction of the standard membrane composite and membranes with varying LTO ratios.

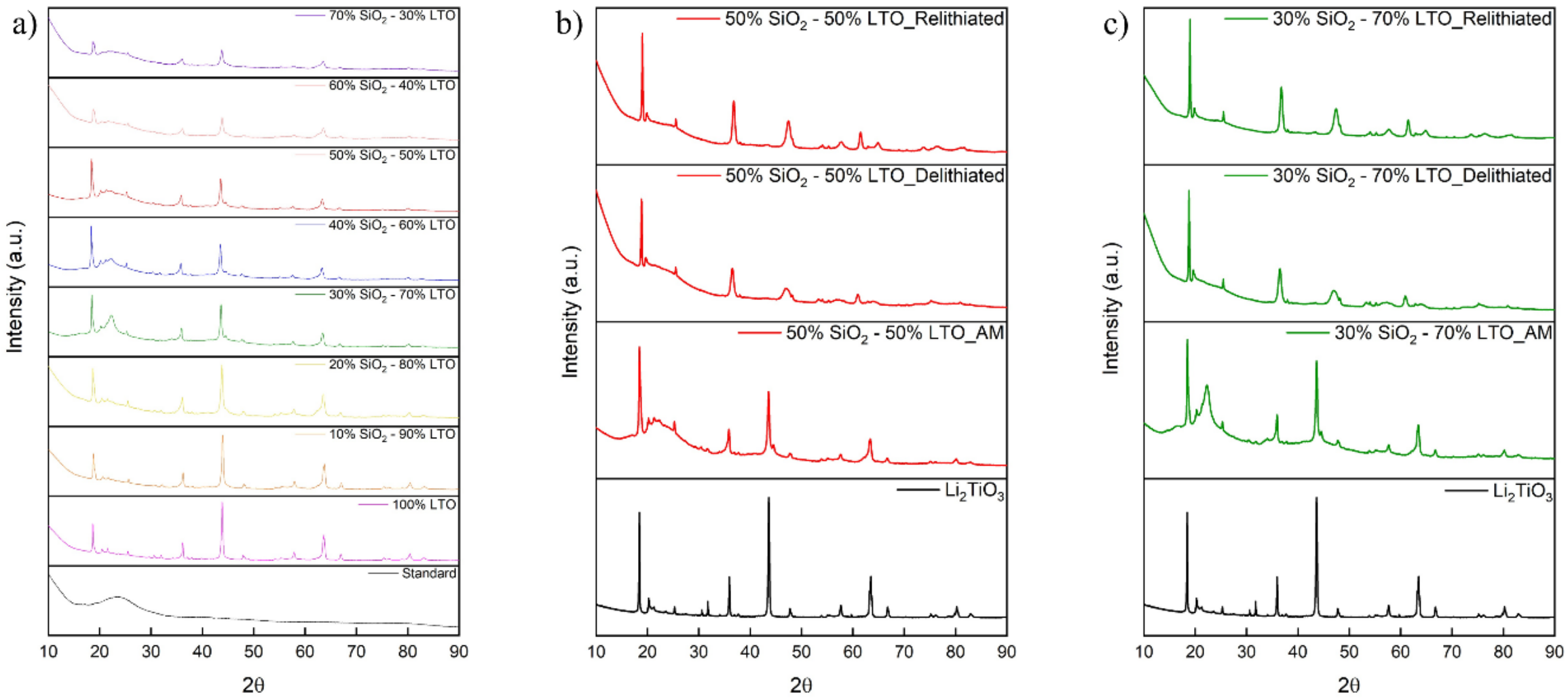

Figure S3. a) X-ray diffraction patterns of the standard membrane and membranes with varying LTO ratios, b) XRD patterns of 50% SiO_2–50% LTO and c) 30% SiO_2–70% LTO in as-made, delithiated, and relithiated states.

S5. Scanning electron microscopy (SEM) and powder X-ray diffraction of the standard membrane composite, its delithiated and relithiated forms

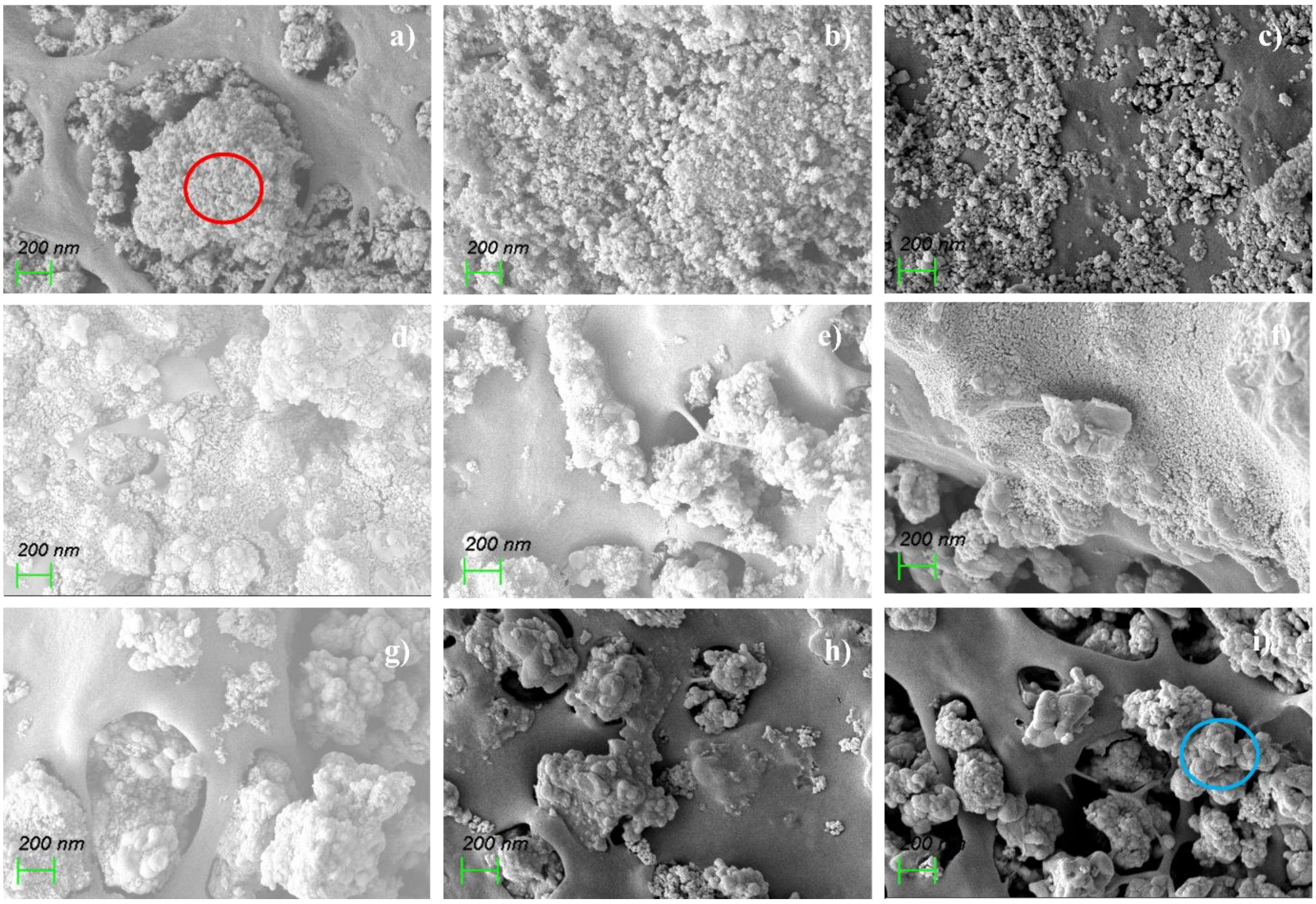

Figure S4. SEM images of (a) standard membrane formulation; (b) 70% SiO_2–30% LTO; (c) 60% SiO_2–40% LTO; (d) 50% SiO_2–50% LTO; (e) 40% SiO_2–60% LTO; (f) 30% SiO_2–70% LTO; (g) 20% SiO_2–80% LTO; (h) 10% SiO_2–90% LTO; and (i) 100% LTO.

SEM micrographs reveal progressive morphological transition with increasing LTO loading. Silica particles are circled in red, and LTO particles in blue. The standard membrane exhibits a continuous polymer matrix with pronounced macrovoids generated during phase inversion. At intermediate LTO contents (40–60 wt%), LTO particles are well dispersed within the PVC/SiO_2 matrix, forming a hierarchical microstructure with visible interparticle voids and retained

macrovoid regions. At higher loadings (≥70 wt%), particle crowding and agglomeration become evident, leading to reduced polymer continuity and densification of the composite structure. These morphological observations are consistent with mercury intrusion porosimetry results, which show a shift toward smaller dominant pore diameters and reduce pore volume.

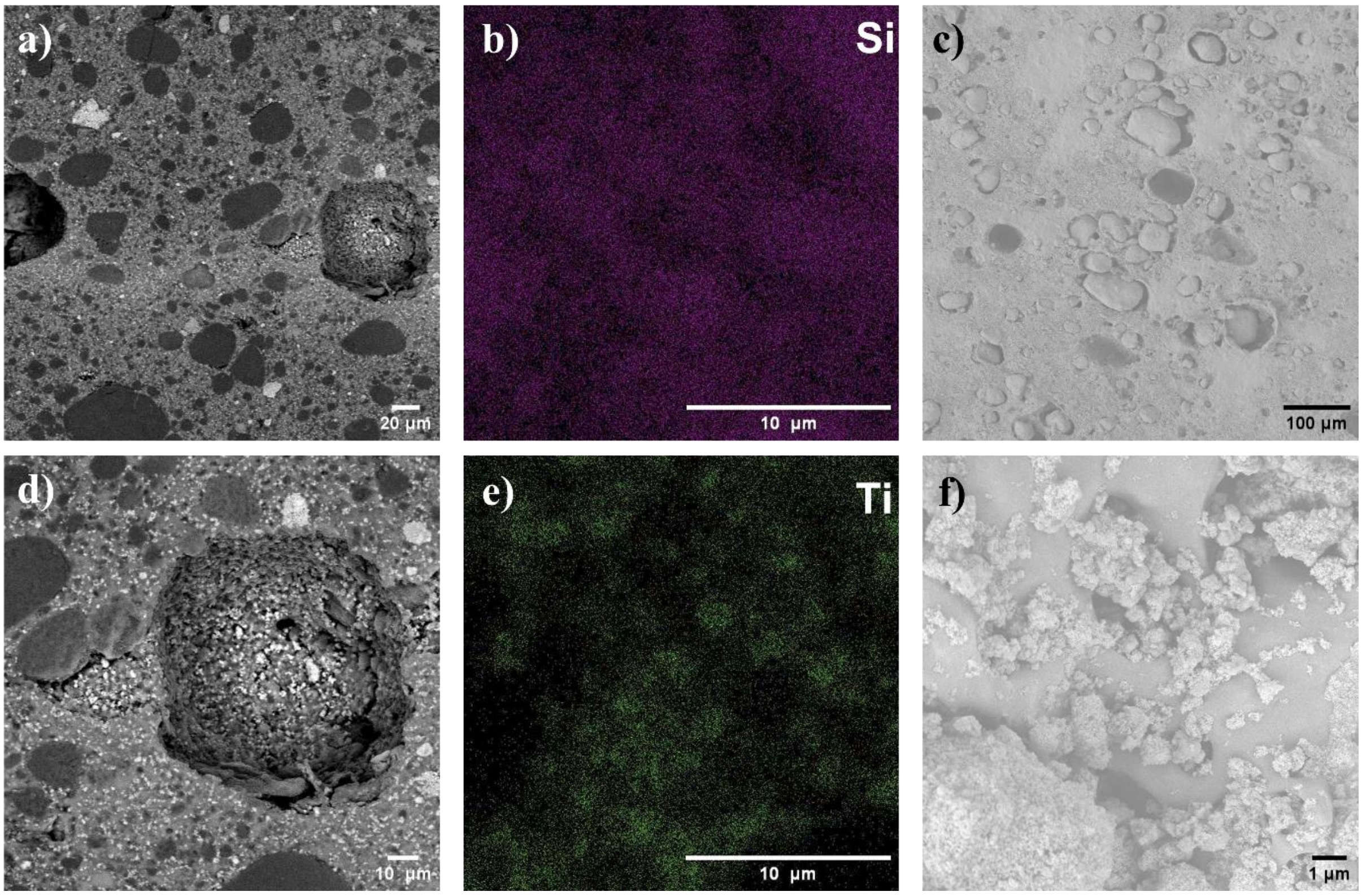

Figure S5. SEM images of the 50% SiO_2–50% LTO membrane: (a, d) cross-sectional views, (b, e) EDS mapping of Si and Ti and (c, f) surface views.

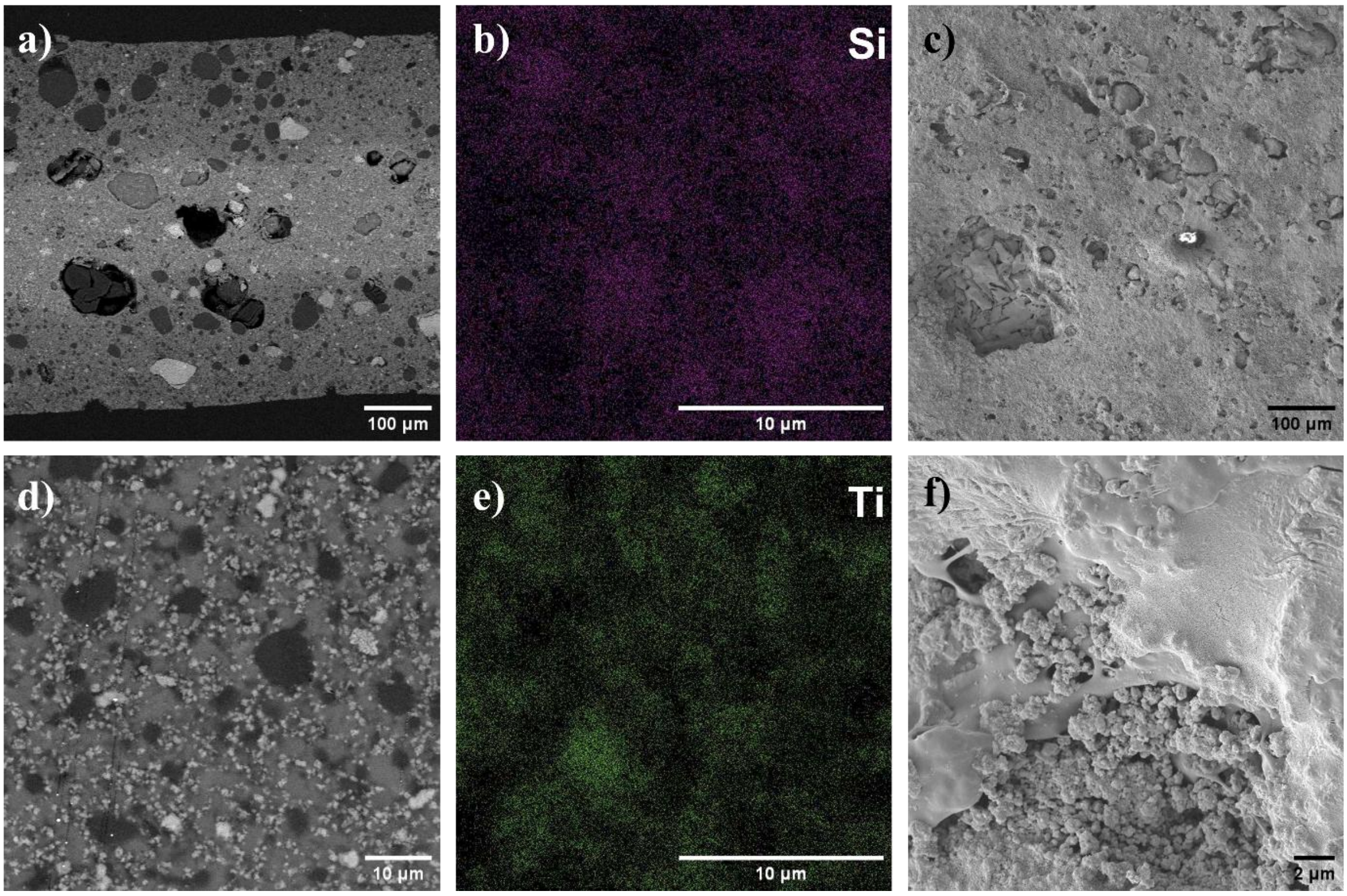

Figure S6. SEM images of the 30% SiO_2–70% LTO membrane: (a, d) cross-sectional views, (b, e) EDS mapping of Si and Ti and (c, f) surface views.

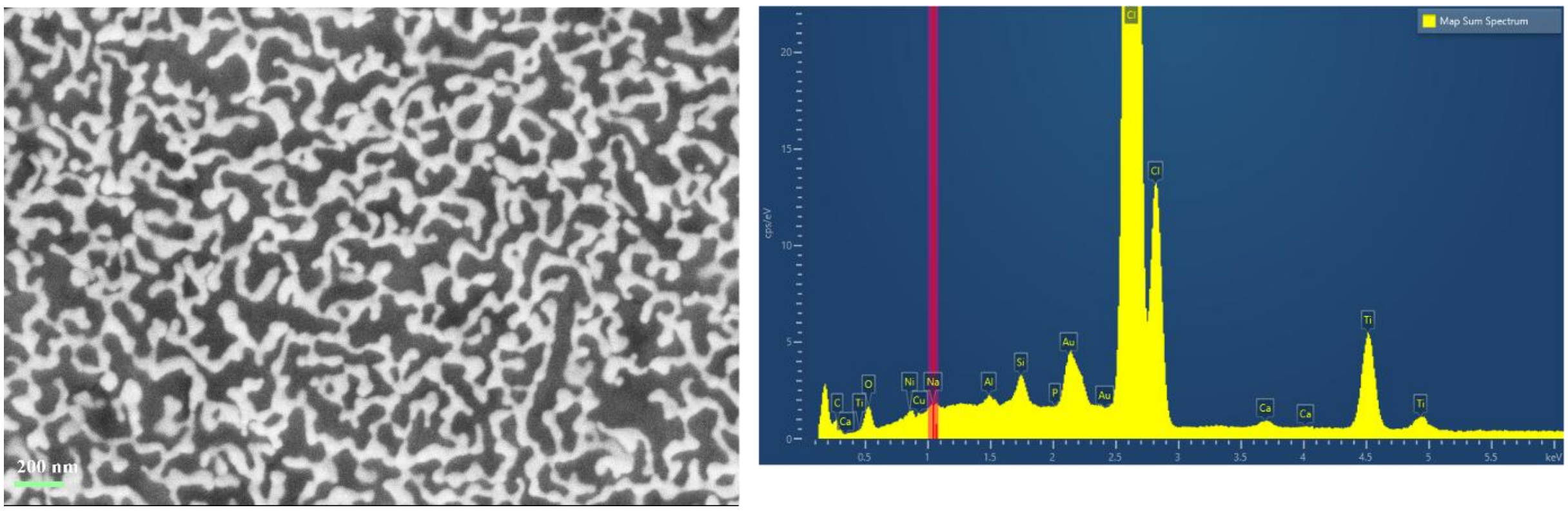

Figure S7. SEM images of particulate deposits recovered from the desorption solution of the 40% SiO_2–60% LTO membrane and EDS map sum spectrum of particulate deposits from the desorption solution of the 40% SiO_2–60% LTO composite membrane confirming the presence of titanium.

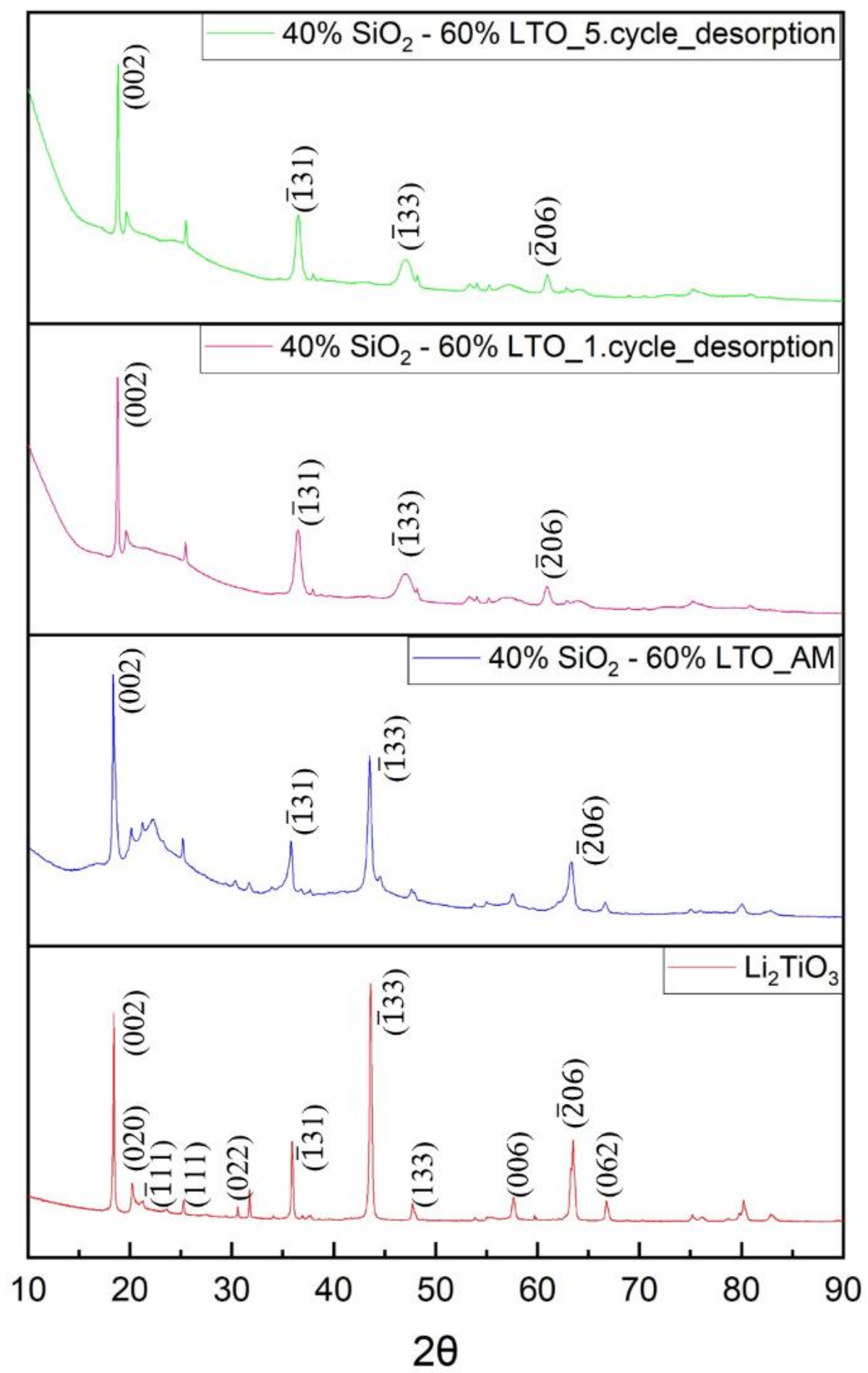

Figure S8. X-ray diffraction patterns of LTO, its membrane composite, the delithiated composite (HTO) after first cycle and dilithiated composite (HTO) after the fifth cycle.

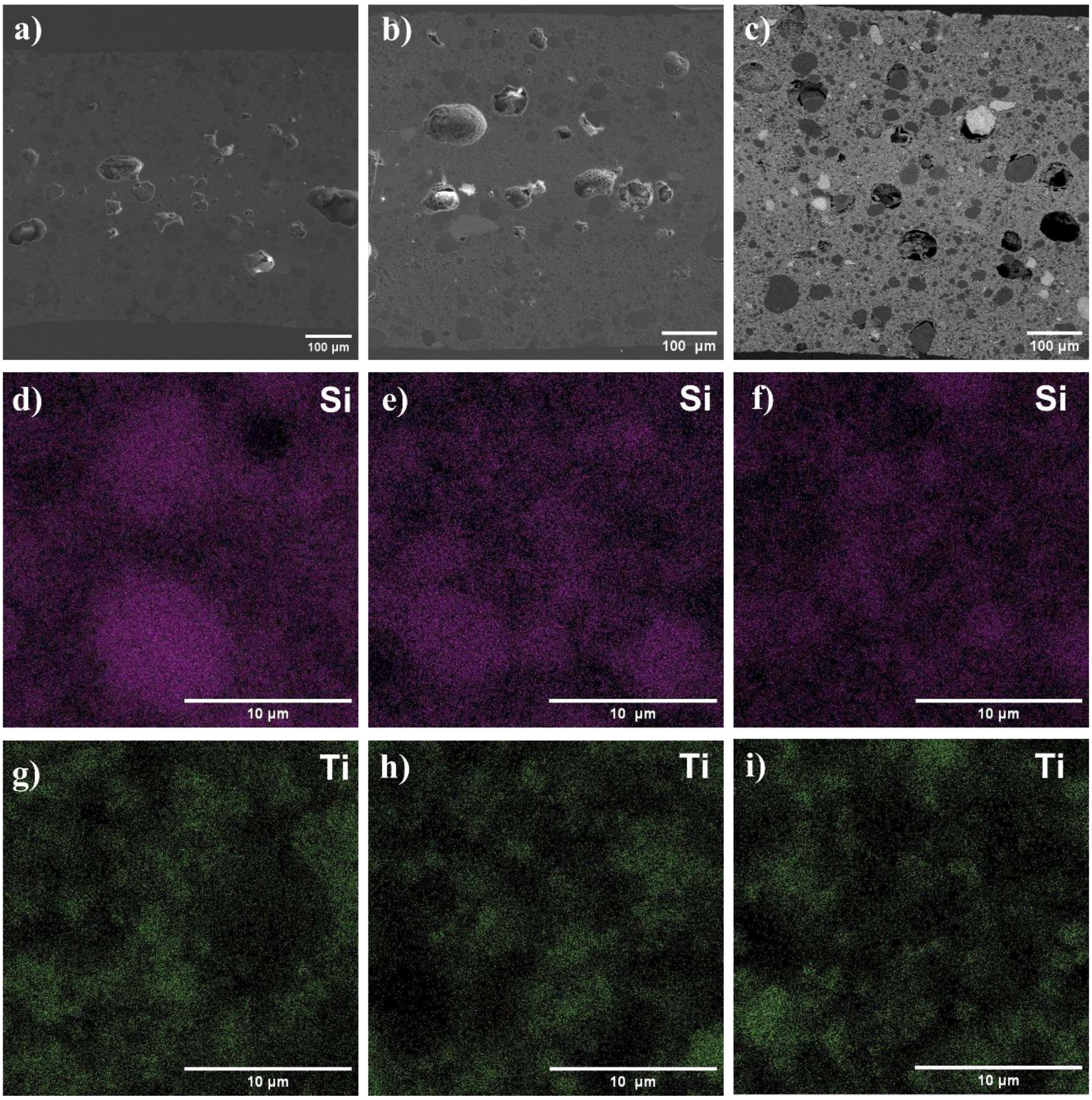

Figure S9. SEM images of the 40% SiO_2–60% LTO membrane: (a, d and g) cross-sectional views and EDS mapping of the as-made membrane, (b, e and h) cross-sectional views and EDS mapping of delithiated membrane and (c, f and i) cross-sectional views and EDS mapping of the relithiated membrane.

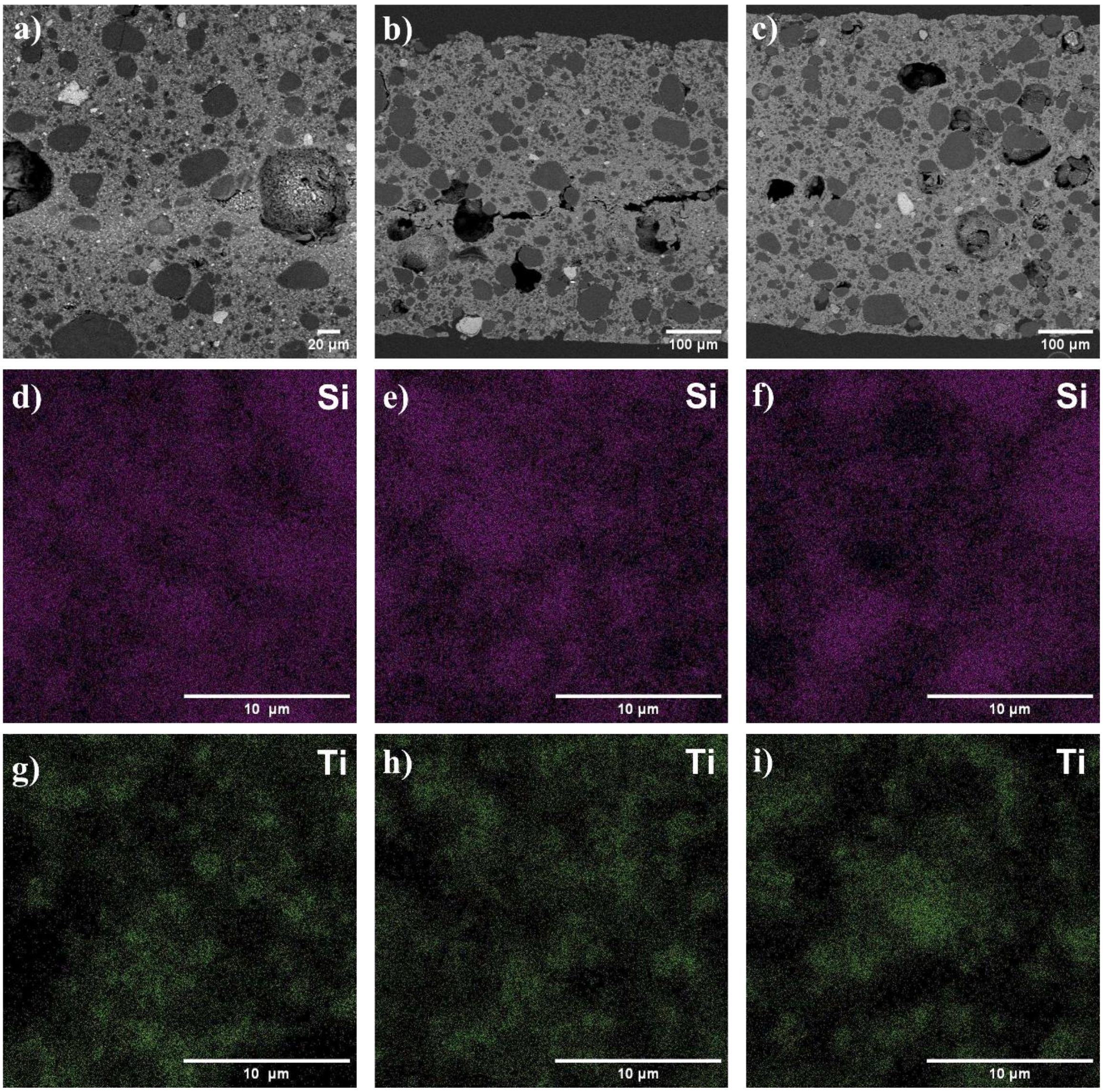

Figure S10. SEM images of the 50% SiO_2–50% LTO membrane: (a, d and g) cross-sectional views and EDS mapping of the as-made membrane, (b, e and h) cross-sectional views and EDS mapping of delithiated membrane and (c, f and i) cross-sectional views and EDS mapping of the relithiated membrane.

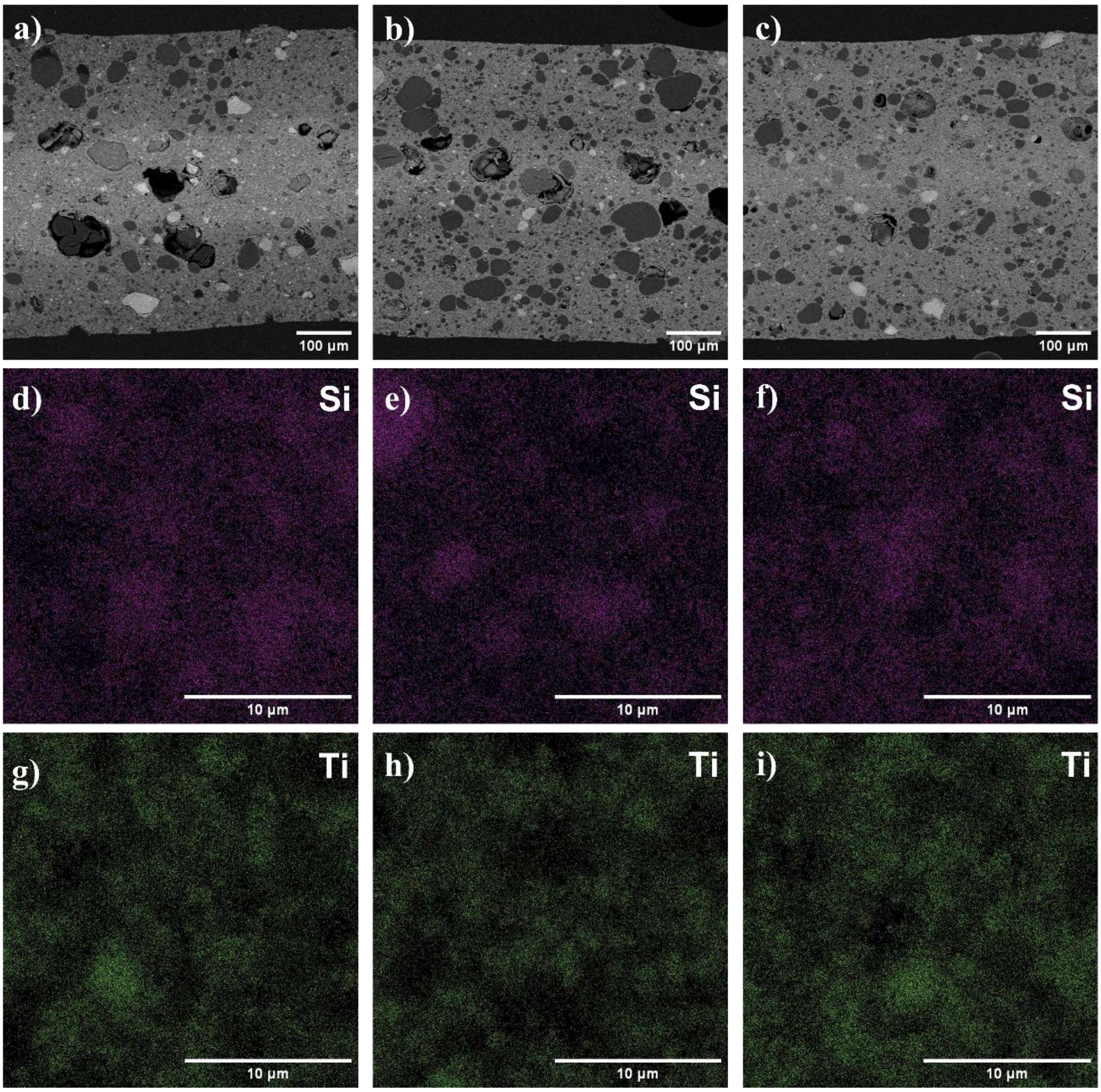

Figure S11. SEM images of the 30% SiO_2–70% LTO membrane: (a, d and g) cross-sectional views and EDS mapping of the as-made membrane, (b, e and h) cross-sectional views and EDS mapping of delithiated membrane and (c, f and i) cross-sectional views and EDS mapping of the relithiated membrane.

S6. Preliminary techno-economic assessment

Table S3. Preliminary techno-economic assesment of the PVC/SiO_2/LTO membrane composite developed in this study.

Parameter	Low	Mid	High	Basis
Membrane composition (as tested)	PVC 44.1 wt%, SiO2 22.4 wt%, Li_2TiO_3 33.5 wt%			Batch: 20.8 g PVC + 10.54 g SiO_2 + 15.81 g LTO (47.15 g total)
Raw material price-PVC	€0.80/kg			Mid-point of manuscript cited range (€0.6-1/kg)
Raw material price-SiO_2	€1.78/kg			Mid-point of manuscript cited range (€0.05-3.5/kg)
Raw material price-Li_2TiO_3	Li_2TiO_3 = €8.60/kg	Li_2TiO_3 = €27.2/kg	Li_2TiO_3 = €86/kg	Manuscript cited range (€8.6-80/kg), mid = geometric mean
Raw material cost per kg membrane	€3.63/kg	€9.86/kg	€29.56/kg	Weighted by composition above
LTO share of raw material cost	79%	92%	98%	Despite being only 33.5 wt% of the membrane
Equilibrium Li^+ adsorption capacity	37 mg g^{-1} HTO (45°C); 25 mg g^{-1} (25°C)			This study, 40% SiO_2 – 60% LTO membrane

Li recovered per kg membrane	12.4 g			37 mg g^{-1} HTO capacity scaled to whole membrane basis
Cost per kg Li recovered	€293	€796	€2385	Raw material cost/Li recovered per kg membrane
Reference market value of Li recovered	~€101/kg contained Li			Battery grade Li_2CO_3
Regeneration reagent cost	~€0.13/kg membrane per cycle			0.2M HCl, bulk industrial pricing
Cumulative Ti^{4+} loss over 5 cycle	41.8% (45°C)			ICP-OES analysis of desorption eluate
Per cycle Ti^{4+} loss trend	9.4% → 6.4%, decelerating			Consistent with detachement of loosely bound particles
Reuse cycles for cost parity with recovered-Li value	~3-24 cycles			Depends on LTO sourcing price

S7. Kinetic parameters and Li^+ extraction efficiencies of three membrane composites

Table S4. Kinetic parameters for Li^+ adsorption on three membrane composites.

Sample	Pseudo-first order			Pseudo-second order		
	q_e (mg g^{-1})	k_1(min^{-1})	r^2	q_e (mg g^{-1})	k_2 (g mg^{-1} min^{-1})	r^2
50% SiO_2 – 50% LTO	32.23	0.01288	0.96	36.82	4.37 x 10^{-4}	0.99
40% SiO_2 – 60% LTO	32.06	0.01628	0.92	36.06	5.83 x 10^{-4}	0.97
30% SiO_2 – 70% LTO	18.33	0.00413	0.96	21.85	2.16 x 10^{-4}	0.99

Table S5. Li^+ extraction efficiency and initial removal rate of PVC/SiO_2/LTO composite membranes with varying LTO content at 45°C (C_0 = 100 mg L^{-1}, S/L = 6 g L^{-1}).

Sample	LTO mass fraction (%)	% extraction, 60 min	% extraction, 24h	Initial rate, 0-15 min (mg L^{-1} min^{-1})
50% SiO_2 – 50% LTO	27.9	28.9	57.6	1.02
40% SiO_2 – 60% LTO	33.5	36.3	73.0	1.62
30% SiO_2 – 70% LTO	39.1	12.6	46.5	0.27

Table S6. Comparison of lithium adsorption capacities of this work with lithium-ion sieve (LIS) adsorbents and membranes reported in the literature.

Paper reference	Composite/Adsorbent type	Initial concentration(c_0)	Adsorption capacity (q_e)
**Wang et al. (2023)[1]**	PVC-LMZO membrane	130 mg L^{-1}	18.33 mg g^{-1}
**Zhu et al. (2014)[2]**	PVC-$H_{1.6}Mn_{1.6}O_4$ Membrane	150 mg L^{-1}	26.64 mg g^{-1}
**Chitrakrar et al. (2014)[3]**	H_2TiO_3 (HTO) Powder	1630 mg L^{-1}	32.6 mg g^{-1}
**Chung, Lawagon et al. (2017)[4]**	LIS/PAN Nanofiber Network ($H_{1.6}Mn_{1.6}O_4$ in polyacrylonitrile)	3-30 mg L^{-1} (real seawater)	16.48 mg g^{-1} (Langmuir max)
**Chung, Lawagon et al. (2019)[5]**	HTO/PAN Nanofiber Composite	70 mg L^{-1}	72.75 mg g^{-1} (Langmuir max)
**Wang et al. (2019)[6]**	Mo-doped Titanium Oxide (HTO) Powde	1800 mg L^{-1}	78 mg g^{-1}
**Zhu et al. (2020)[7]**	Anatase-derived H_2TiO_3 (HTO) Powder	200-400 mg L^{-1}	34.2 mg g^{-1}

**Chung, Lawagon et al. (2016)**[8]	H_2TiO_3 (HTO) Powder	0.7-7000 mg L^{-1}	94.5 mg g^{-1} (at 7000 mg/L)
**Zhang et al.(2023)**[9]	HTO/RGO-TA Membrane Electrode (PVA binder)	303 mg L^{-1}	25.2 mg g^{-1}
**This work**	PVC/SiO_2/LTO membrane composite	100 mg L^{-1}	37 mg g^{-1}

REFERENCES

(1) Wang, L.; Li, L. Preparation of PVC-LMZO Membrane and Its Lithium Adsorption Performance from Brine. *Desalination* **2023**, *561*. https://doi.org/10.1016/j.desal.2023.116689

(2) Zhu, G.; Wang, P.; Qi, P.; Gao, C. Adsorption and Desorption Properties of Li+ on PVC-H1.6Mn1.6O4 Lithium Ion-Sieve Membrane. *Chem. Eng. J.* **2014**, *235*, 340–348. https://doi.org/10.1016/j.cej.2013.09.068

(3) Ramesh Chitrakar. Lithium Recovery from Salt Lake Brine by H2TiO3. *Dalton Trans.* **2014**, *43* (23), 8933–8939.

(4) Chung, W. J.; Torrejos, R. E. C.; Park, M. J.; Vivas, E. L.; Limjuco, L. A.; Lawagon, C. P.; Parohinog, K. J.; Lee, S. P.; Shon, H. K.; Kim, H.; Nisola, G. M. Continuous Lithium Mining from Aqueous Resources by an Adsorbent Filter with a 3D Polymeric Nanofiber Network Infused with Ion Sieves. *Chem. Eng. J.* **2017**, *309*, 49–62. https://doi.org/10.1016/j.cej.2016.09.133

(5) Lawagon, C. P.; Nisola, G. M.; Cuevas, R. A. I.; Kim, H.; Lee, S. P.; Chung, W. J. Development of High Capacity Li+ Adsorbents from H2TiO3/Polymer Nanofiber Composites: Systematic Polymer Screening, Characterization and Evaluation. *J. Ind. Eng. Chem.* **2019**, *70*, 124–135. https://doi.org/10.1016/j.jiec.2018.10.003

(6) Wang, S.; Zhang, M.; Zhang, Y.; Zhang, Y.; Qiao, S.; Zheng, S. High Adsorption Performance of the Mo-Doped Titanium Oxide Sieve for Lithium Ions. *Hydrometallurgy* **2019**, *187*, 30–37. https://doi.org/10.1016/j.hydromet.2019.05.004

(7) Li, X.; Chao, Y.; Chen, L.; Chen, W.; Luo, J.; Wang, C.; Wu, P.; Li, H.; Zhu, W. Taming Wettability of Lithium Ion Sieve via Different TiO2 Precursors for Effective Li Recovery from Aqueous Lithium Resources. *Chem. Eng. J.* **2020**, *392*. https://doi.org/10.1016/j.cej.2019.123731

(8) Lawagon, C. P.; Nisola, G. M.; Mun, J.; Tron, A.; Torrejos, R. E. C.; Seo, J. G.; Kim, H.; Chung, W. J. Adsorptive Li+ Mining from Liquid Resources by H2TiO3: Equilibrium, Kinetics, Thermodynamics, and Mechanisms. *J. Ind. Eng. Chem.* **2016**, *35*, 347–356. https://doi.org/10.1016/j.jiec.2016.01.015

(9) Zhang, J.; Cheng, Z.; Qin, X.; Gao, X.; Yun, R.; Xiang, X. Bifunctional Modification Enhances Lithium Extraction from Brine Using a Titanium-Based Ion Sieve Membrane Electrode. *ACS Appl. Mater. Interfaces* **2023**, *15* (24), 29586–29596. https://doi.org/10.1021/acsami.3c04682
